# Balanced Codon Usage Optimizes Eukaryotic Translational Efficiency

**DOI:** 10.1371/journal.pgen.1002603

**Published:** 2012-03-29

**Authors:** Wenfeng Qian, Jian-Rong Yang, Nathaniel M. Pearson, Calum Maclean, Jianzhi Zhang

**Affiliations:** 1 Department of Ecology and Evolutionary Biology, University of Michigan, Ann Arbor, Michigan, United States of America; 2 Key Laboratory of Gene Engineering of the Ministry of Education, State Key Laboratory of Biocontrol, School of Life Sciences, Sun Yat-sen University, Guangzhou, China; Fred Hutchinson Cancer Research Center, United States of America

## Abstract

Cellular efficiency in protein translation is an important fitness determinant in rapidly growing organisms. It is widely believed that synonymous codons are translated with unequal speeds and that translational efficiency is maximized by the exclusive use of rapidly translated codons. Here we estimate the *in vivo* translational speeds of all sense codons from the budding yeast *Saccharomyces cerevisiae*. Surprisingly, preferentially used codons are not translated faster than unpreferred ones. We hypothesize that this phenomenon is a result of codon usage in proportion to cognate tRNA concentrations, the optimal strategy in enhancing translational efficiency under tRNA shortage. Our predicted codon–tRNA balance is indeed observed from all model eukaryotes examined, and its impact on translational efficiency is further validated experimentally. Our study reveals a previously unsuspected mechanism by which unequal codon usage increases translational efficiency, demonstrates widespread natural selection for translational efficiency, and offers new strategies to improve synthetic biology.

## Author Summary

Although an amino acid can be encoded by multiple synonymous codons, these codons are not used equally frequently in a genome. Biased codon usage is believed to improve translational efficiency because it is thought that preferentially used codons are translated faster than unpreferred ones. Surprisingly, we find similar translational speeds among synonymous codons. We show that translational efficiency is optimized by a previously unknown mechanism that relies on proportional use of codons according to their cognate tRNA concentrations. Our results provide important molecular details of protein translation, answer why codon usage is unequal, demonstrate widespread natural selection for translational efficiency, and can guide designs of synthetic genomes and cells with efficient translation systems.

## Introduction

Eighteen of the 20 amino acids are each encoded by two or more synonymous codons in the standard genetic code, yet the synonymous codons are often used unequally in a genome. Such codon usage bias (CUB) has been extensively documented in all three domains of life [1]–[3]. Within a genome, highly expressed genes tend to have stronger CUB than lowly expressed ones [4], and the codons preferentially used in highly expressed genes of a species are referred to as *preferred codons*.

Although codon usage is clearly determined by the joint actions of mutation, drift, and selection [5]–[6], the fitness benefit of CUB is less clear. There are two prevailing, non-mutually exclusive, hypotheses on the selective utility of CUB: accuracy and efficiency of protein translation [6]. The translational accuracy hypothesis asserts that different synonymous codons have different probabilities of mistranslation, and that the use of accurately translated codons is beneficial because mistranslation reduces the number of functional molecules, wastes energy, and/or induces cytotoxic protein misfolding. Unequivocal evidence for this hypothesis exists [7]–[10].

By contrast, the translational efficiency hypothesis lacks direct evidence. This hypothesis holds that different synonymous codons are translated at different speeds, and that faster translation is beneficial because it minimizes ribosome sequestering and so helps alleviate ribosome shortage [5], [11]–[12]. The relevance of ribosome shortage is evident from the findings that most ribosomes are actively engaged in translation during rapid cell growth [13]–[14] and that ribosome concentration increases with the rate of cell growth [15]. An important observation invoked to support the efficiency hypothesis is that cognate tRNAs of preferred codons tend to have higher cellular concentrations (or more gene copies) than those of unpreferred codons [4], [16], which may allow faster translation of preferred codons than unpreferred codons. While results from several earlier studies are consistent with this hypothesis [12], [17], these studies do not exclude the possibility that the observed differences in activity or fitness caused by synonymous mutations are entirely due to CUB's influence on translational accuracy (see Discussion). Here we directly test the efficiency hypothesis and its presumed underlying mechanism.

## Results

### Estimating *in vivo* translational speeds

**Figure 1:**
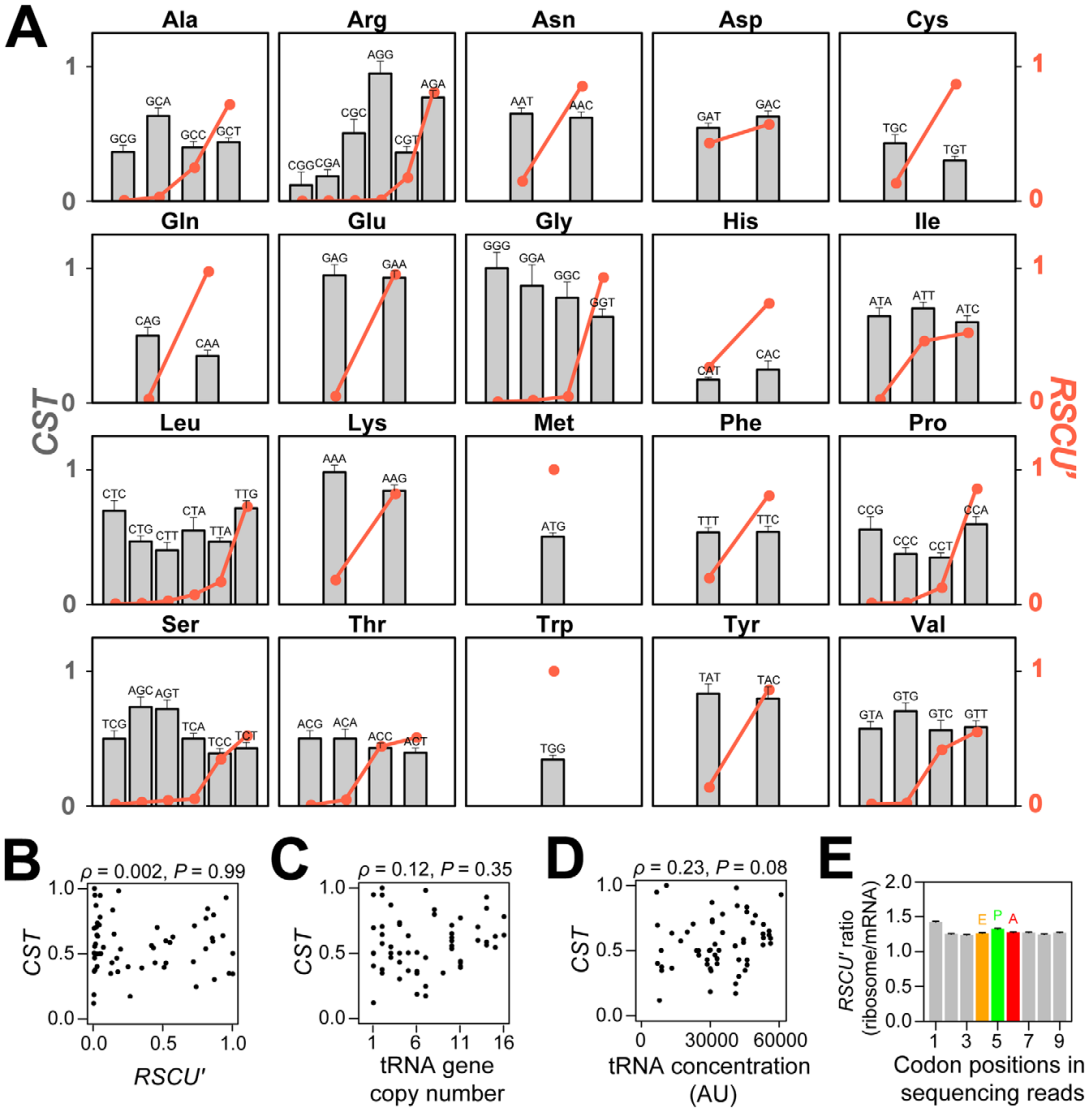
Relative codon selection times (*CST*s) in wild-type yeast cells in rich media. (A) *CST* (grey bars) and *RSCU'* (orange dots) of each sense codon. *CST*s are rescaled such that the maximal observed value is 1. Error bars show one standard error, estimated by the bootstrap method. No significant negative correlation between *CST* and (B) *RSCU'*, (C) tRNA gene copy number, or (D) tRNA concentration. Spearman's rank correlation coefficients (ρ) and associated *P* values are presented above each panel. The *P* value in (B) is calculated by a permutation test because of the non-independence among *RSCU'* values of synonymous codons. (E) No dip in *RSCU'* at the ribosomal A site, compared to P, E, and other neighboring sites. Geometric means of *RSCU'* is calculated at each codon position (as in the calculation of *CAI*) for ribosome profiling sequencing reads and mRNA sequencing reads, respectively; the ratio at each position is presented. Error bars show one standard error estimated by bootstrapping sequencing reads 1000 times.

The translational efficiency hypothesis assumes that synonymous codons have different translational speeds, caused by disparities in codon selection time (*CST*), the time needed for ribosomal A site to find the cognate ternary complex of aminoacylated tRNA+eEF-1α+GTP. To test this proposition, we took advantage of a genome-wide ribosome profiling study of *Saccharomyces cerevisiae* that surveyed ribosome-protected mRNA fragments at a nucleotide resolution in a cell population at a given moment by Illumina deep sequencing [18]. Because the probability that a codon is docked at the A site is proportional to its *CST*, we estimated the relative *CST*s of all 61 sense codons (Figure 1A) by the ratio of the observed codon frequencies at the A site in the ribosome profiling data and the expected codon frequencies estimated from mRNA-Seq data generated under the same condition in the same experiment (Figures S1, S2, S3; see Materials and Methods). The standard errors of the *CST* estimates, measured by bootstrapping genes from the original datasets, are on average 12% of the *CST* estimates (Figure 1A), indicating that our *CST* estimates are overall quite precise.

CUB is commonly measured by the *relative synonymous codon usage* (*RSCU*), defined by the frequency of a codon relative to the average frequency of all of its synonymous codons in a set of highly expressed genes [19]. To compare the usage of all 61 sense codons, we also use *RSCU'*, which is the proportion of use of a given codon among synonymous choices in a set of highly expressed genes (see Materials and Methods). Another commonly used measure of CUB is the *codon adaptation index* (*CAI*) [20], which is calculated for a gene, and measures its usage of high-*RSCU* codons (see Materials and Methods). The greater the *CAI*, the more prevalent are preferred codons in the gene.

Contrary to the widely held presumption that preferred codons are translated faster than unpreferred codons, no significant negative correlation between *RSCU'* and *CST* was observed among the 61 sense codons (Figure 1B). It is also believed that codons with abundant cognate tRNAs tend to have low *CST*s. Because tRNA gene copy number and tRNA concentration are highly positively correlated [21]–[22], the former is often used as a proxy of the latter. However, neither tRNA gene copy number (Figure 1C) nor tRNA concentration (Figure 1D) correlates negatively with *CST*. Because codons and tRNAs do not have one-to-one correspondence, in the foregoing analysis, we considered the best-matching tRNA species for each codon. This codon-tRNA relationship has been shown to be more accurate than the wobble rule, at least in yeast [22].

We also examined each amino acid separately. Among the 18 amino acids with at least two codons, 12 (Ala, Asn, Cys, Gln, Glu, Gly, Ile, Lys, Ser, Thr, Tyr, and Val) showed a negative correlation between *RSCU'* and *CST*, while 6 (Arg, Asp, His, Leu Phe, and Pro) showed a positive correlation, when statistical significance of the correlation was not required (Figure 1A). The number of negative correlations is not significantly more than the chance expectation of 9 (*P* = 0.12, one-tail sign test).

Using the standard errors of the *CST* estimates for the foregoing 18 amino acids (Figure 1A), we tested whether the *CSTs* are significantly different between the synonymous codon with the highest *RSCU'* and that with the lowest *RSCU'*. After the control for multiple testing by the Bonferroni correction, only two amino acids showed significant differences. The highest-*RSCU'* codon has a lower *CST* than the lowest-*RSCU'* codon for glycine (nominal *P* = 0.002), while the opposite is true for arginine (nominal *P*<0.001). Our results are robust to different multiple-testing corrections, as no other amino acids show a nominal *P*<0.01. Furthermore, when *RSCU'* is not considered, arginine is the only amino acid for which synonymous codons show significant heterogeneity in *CST* at the 5% significance level after the correction for multiple testing. Following an earlier study [1], we also tried defining preferred codons without using gene expression data, but the results are not different (Figure S4). The overall lack of a significant negative correlation between *CST* and synonymous codon usage is real rather than an artifact of imprecise *CST* estimation, because the standard errors of *CSTs* are quite small (Figure 1A) and *CST*s of several nonsynonymous codons differ significantly from one another (see below).

To validate the above findings, we also directly compared *RSCU'* values of individual codon positions of Illumina reads from the ribosome profiling data, without estimating *CST*s. If unpreferred codons are translated more slowly and therefore stay at the ribosomal A site longer than preferred codons, codons at the A site should have a lower *RSCU'* on average than its neighboring sites of the same read, after the correction of sequencing bias by mRNA-Seq data. However, we observed no dip in *RSCU'* at the A site (Figure 1E). We further calculated, within each gene, the ratio between the frequency of preferred codons and that of unpreferred codons at the ribosome A site of Illumina reads from the ribosome profiling data, after correction by mRNA-Seq. This ratio is expected to be 1 if preferred and unpreferred codons are translated equally fast. Indeed, after combining the ratio for all amino acids and all genes using the Mantel-Haenszel procedure [23], we found the overall ratio to be 0.984, not significantly different from 1 (*P* = 0.21, two-tail χ^2^ test).

### Optimal codon usage under tRNA shortage

The above findings are puzzling, because the first step in the interaction between tRNA and mRNA is non-specific [24] and the relative waiting time for the cognate tRNA to arrive at the ribosome A site is expected to be inversely proportional to the relative concentration of the cognate tRNA. It was also reported that *CST* is the rate-limiting step in translational elongation [25]. The only plausible explanation of similar *CST*s among synonymous codons is that, in wild-type yeast cells for which the ribosome profiling was conducted, available cognate tRNAs for translating synonymous codons have effectively the same concentration.

In rapidly growing yeast, ∼80% of total RNA is rRNA and ∼15% is tRNA [15]. The mean length of yeast tRNAs is ∼72 nucleotides and the total length of rRNAs per ribosome is 5469 nucleotides [15]. Thus, the number of tRNA molecules per cell is approximately (15%/72)/(80%/5469) = 14.2 times the number of ribosomes per cell, substantially exceeding the expected ratio of two tRNAs per active ribosome (at A and P sites, respectively) if tRNA recharging and diffusion is instantaneous.

In reality, however, tRNA recycling takes time and thus cannot be ignored. Each tRNA, after completing its job of transferring an amino acid to the elongating peptide and then exiting the ribosomal E site, needs to be recharged with the cognate amino acid and then with eEF-1α+GTP to form a ternary complex before it can be reused in translation. It has been estimated that each ribosome translates ∼32.6 codons per second in yeast [26]. This implies that on average a tRNA molecule needs to be used 32.6/14.2 = 2.3 times per second, or once every 0.44 second. It is possible that the time for ternary complexes to form and diffuse to ribosomal A site is a substantial fraction of 0.44 second, so that the local concentration of ternary complexes is much lower than the total tRNA concentration. A recent study reported that consecutive synonymous codons in an mRNA tend to use the same tRNA and proposed that this codon choice is beneficial because a tRNA does not diffuse far from the ribosome after exiting its E site and is reused for translating the next synonymous codon when the ternary complex is formed again [27]. This observation and its explanation strongly implies that the local concentration of ternary complexes is low; otherwise, the addition of one cognate tRNA molecule among on average 20 tRNAs (because identical amino acids are expected to be on average 20 residues apart) cannot significantly increase the relative concentration of the cognate tRNA around the ribosome. Based on available information in *E. coli*, we calculated that the physiological concentration of ternary complexes is only ∼4.3% of the total concentration of tRNAs and ∼22% of the concentration of ribosomes (see Materials and Methods). These observations strongly support our hypothesis that available tRNA is in shortage during translation. Consistent with our hypothesis, total tRNA concentrations increase with the rate of cell growth in *E. coli* [28] and tRNA gene copy number increases with the shortening of the minimal generation time across species [29].

Under tRNA shortage, the optimal usage of synonymous codons in minimizing the total *CST* (i.e., maximizing translational efficiency) is to use isoaccepting tRNAs in proportion to their concentrations (see Materials and Methods). That is, *p_i_* = *q_i_*, where *p_i_* is the relative usage of the *i*th synonymous codon of an amino acid (Σ*p_i_* = 1) and *q_i_* is the relative concentration of the corresponding tRNA (Σ*q_i_* = 1). Under this codon usage, available cognate tRNAs of synonymous codons have equal concentrations and synonymous codon selection times become identical (see Materials and Methods). We will refer to this theoretical optimal codon usage under tRNA shortage as the proportional rule. The proportional rule is not predicted by other models. For example, without tRNA shortage, two optimal solutions in minimizing the total *CST* exist. When codon usage is fixed, isoaccepting tRNA concentrations should follow 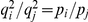, which is referred to as the square rule [30]–[31]. When tRNA concentrations are fixed, only the codon corresponding to the most abundant tRNA species should be used [30], which is referred to as the truncation rule.

**Figure 2:**
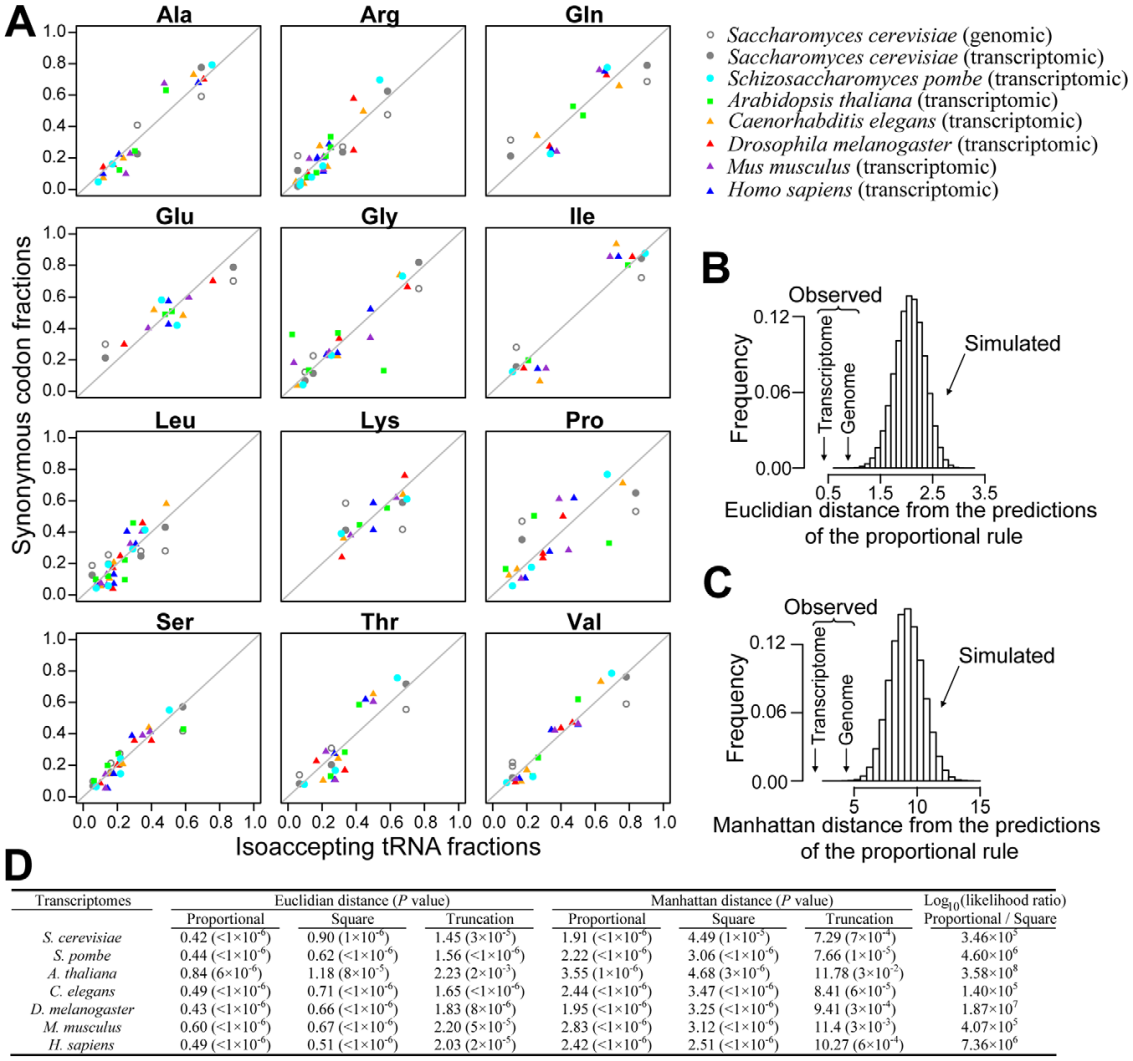
Synonymous codons are used in proportion to cognate tRNA concentrations. (A) Relative uses of synonymous codons in the transcriptomes of seven model eukaryotes are compared to the relative concentrations of cognate tRNAs measured from gene copy numbers, for the 12 amino acids that have at least two isoaccepting tRNA species. For comparison, genomic synonymous codon usage in *S. cerevisiae* is also presented. The diagonal line shows the predicted proportional relationship between tRNA concentrations and cognate codon uses that maximizes translational efficiency under tRNA shortage. (B) Euclidian and (C) Manhattan distances between the observed synonymous codon usage in *S. cerevisiae* and the prediction by the proportional rule are significantly smaller than chance expectations. Euclidian and Manhattan distances are defined by 
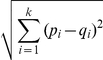
 and 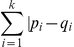, respectively, where *p_i_* and *q_i_* are codon and cognate tRNA fractions, respectively, and *k* is the number of different tRNA species for the amino acid concerned. The chance expectations are shown by the frequency distributions of the distances under uniformly random codon usage, determined from 10^6^ simulations. (D) Euclidian and Manhattan distances between the observed synonymous codon usage and the predictions under the proportional rule, square rule, and truncation rule, respectively. *P* values indicate the probability that a distance generated by random codon usage is smaller than the observed distance, determined by 10^6^ simulations. Log_10_(likelihood ratio) measures the likelihood of the proportional rule, relative to the square rule, given the actual codon usage.

To test if the actual codon usage of yeast follows the proportional rule, we examined the 12 amino acids that are each translated by at least two tRNA species in yeast. For each amino acid, the relative transcriptomic usage of a codon among synonymous codons (i.e., *p_i_*) is quite close to the relative gene copy number of its cognate tRNA among isoaccepting tRNAs (i.e., *q_i_*), as predicted by the proportional rule (Figure 2A). We measured the Euclidian (Figure 2B) and Manhattan (Figure 2C) distances in synonymous codon usage from the observed values to those predicted by the proportional rule, and found these distances significantly shorter than expected by chance (Figure 2B–2D; Table S1; see Materials and Methods). Not surprisingly, genomic codon usage fits the proportional rule less well than the transcriptomic codon usage (Figure 2A), reflected by greater distances from the predicted values (Figure 2B, 2C).

The better fitting of the transcriptomic codon usage to the proportional rule than to the square rule and truncation rule can be seen from a comparison of the distances under these three models (Figure 2D). We also compared the likelihood of the three models, given the observed codon usage (Figure 2D). The proportional model has a much higher log_10_(likelihood) than the square model. Because the likelihood of the truncation model is 0, this model is much worse than the other two models. The same conclusions are reached for the transcriptomic codon usage of all other model eukaryotes we examined (Figure 2A, 2D).

In the above analysis, we combined synonymous codons that are recognized by the same tRNA species (referred to as iso-synonymous codons). Because the relative usage of such iso-synonymous codons does not affect the relative usage of isoaccepting tRNAs, it presumably does not affect translational efficiency. Nonetheless, iso-synonymous codons are not used equally, and factors other than translational efficiency (e.g., translational accuracy) may be at work (Table S2).

### Codon–tRNA imbalance reduces translational efficiency

The observation of similar *CST*s among synonymous codons and the empirical validation of the proportional rule strongly support the following model that includes three elements: (1) available tRNAs are in shortage during translation, (2) translational efficiency is optimized in nature by balanced codon usage according to tRNA concentrations, and (3) synonymous codons are translated with similar speeds under the codon-tRNA balance. Our model predicts reduced translational efficiency due to ribosome sequestering when the codon-tRNA balance is broken. It further predicts lower efficiency under exclusive use of preferred codons than balanced use of preferred and unpreferred codons.

**Figure 3:**
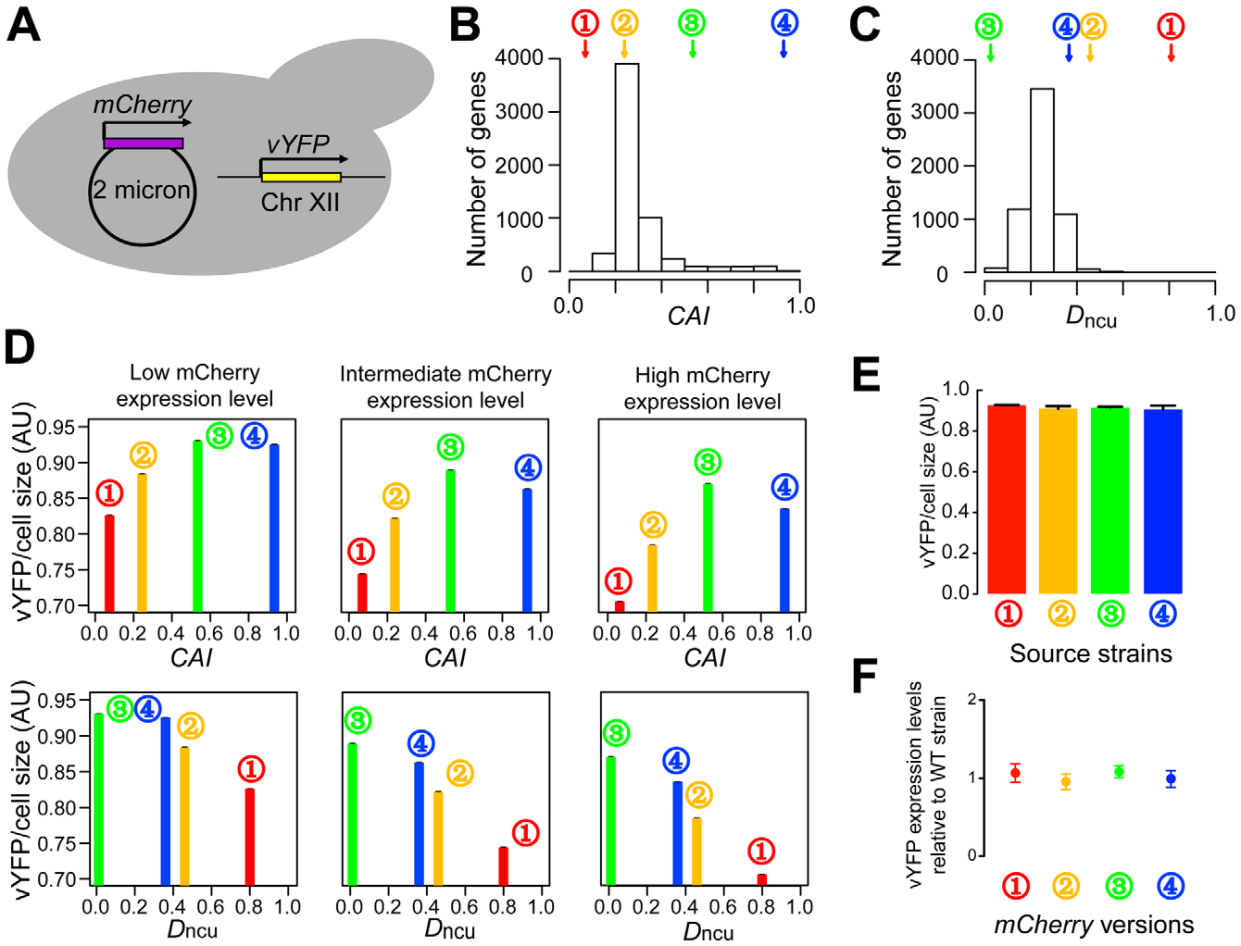
Experimental evidence for the impact of codon usage imbalance on translational efficiency. (A) Experimental design for examining the impact of mCherry expression on the expression of the reporter vYFP. An *mCherry* gene is constitutively expressed from a 2-micron plasmid in *S. cerevisiae*, whereas *vYFP* is constitutively expressed from Chromosome XII. Four different synonymous versions of *mCherry* are compared. (B) The codon adaptation indices (*CAI*s) of the four synonymous *mCherry* sequences (circled numbers), in comparison to *CAI*s of all *S. cerevisiae* genes. (C) Values of distance to native codon usage of yeast (*D*_ncu_) for the four *mCherry* sequences, in comparison to that of all *S. cerevisiae* genes. (D) Relationship between vYFP expression and the *CAI* or *D*_ncu_ of *mCherry*, when the mCherry expression is controlled for. A finer control of mCherry expression is presented in Figure S6, where cells of the low, intermediate, and high mCherry expressions defined here are each subdivided into 5 bins. Error bars, which are barely seen, show one standard error. (E) vYFP expressions in the four strains after the removal of the plasmids that carry *mCherry*. Error bars show one standard error. (F) *vYFP* mRNA levels of the four strains relative to that of the wild-type strain, which does not carry *mCherry*. The mean expressions from three biological replications and the standard errors are presented.

We experimentally tested the above predictions by quantifying the cellular efficiency in translation, represented by the protein expression of a reporter gene, under different levels of codon-tRNA imbalance induced by the expression of another gene. Unlike previous studies [12], [17], our separation of the inducer and reporter allows the distinction among several potential mechanisms of CUB's impact on protein expression. We inserted our reporter gene, the *Venus* yellow fluorescent protein (vYFP) gene controlled by the *GPD* promoter, into Chromosome XII of a haploid strain of *S. cerevisiae* (Figure 3A). We then designed four synonymous sequences encoding another fluorescent protein, mCherry, as our inducer (Figure S5). The four *mCherry* sequences, named *mCherry*-1, 2, 3, and 4, cover the entire range of *CAI* of native yeast genes (Figure 3B). We developed an index, distance to native codon usage (*D*_ncu_), to measure the difference between the codon usage of a (heterologous) gene and the overall codon usage of the host cell, which is proportional to tRNA concentrations (see Materials and Methods). The four *mCherry* versions also span a large range of *D*_ncu_ (Figure 3C) and show different degrees of codon-tRNA imbalance for individual amino acids (Figure S6). Other than synonymous codon usage, the four *mCherry* versions are nearly identical: they encode the same protein sequence, have similar G+C content (42–44%), and have identical sequences in the first 56 nucleotides of the coding region, because this region may affect the level of protein expression [12], [32]–[33]. Each *mCherry* gene is expressed from a constitutive and strong promoter on a high-copy-number plasmid (see Materials and Methods). The four plasmids were separately transformed to yeast cells carrying the vYFP reporter gene (Figure 3A). Our model predicts that the higher the *D*_ncu_ of *mCherry*, the lower the vYFP expression.

The four yeast strains were grown in rich media to the log phase, and the expression levels of vYFP and mCherry proteins were inferred from their fluorescent signals, which were simultaneously measured for each cell by fluorescence-activated cell scanning of at least 300,000 cells. We found mCherry expression levels to be significantly different among the four strains (see Materials and Methods). Within each strain, expression levels of mCherry and vYFP are negatively correlated among cells (see Materials and Methods). Hence, the expressions of vYFP cannot be directly compared among strains. Instead, we separated the cells of each strain into three bins on the basis of mCherry expression and then compared vYFP expressions among the four strains for cells with similar mCherry expressions (Figure 3D). We found that, across the range of mCherry expressions shared by the four strains, the higher the *D*_ncu_ of *mCherry*, the lower the expression of vYFP (Figure 3D). Furthermore, the vYFP expression-level difference among the strains increases with the mCherry expression level (Figure 3D). Of special interest is the comparison between *mCherry*-3 and *mCherry*-4, which clearly shows that it is a low *D*_ncu_ rather than a high *CAI* that enhances translational efficiency (Figure 3D). A multivariate regression analysis of all cells from the four strains further demonstrated that *D*_ncu_ is significantly more important than *CAI* in explaining the variation of the vYFP signal (*P*<0.001).

The above results were not due to different random mutations fixed in the genomes of the four strains during our experiments, because the vYFP signals were not significantly different among the strains upon removal of the plasmids (Figure 3E). We also sequenced the entire plasmid DNA from each strain and found no mutation. Using quantitative polymerase chain reaction, we further verified that the *vYFP* mRNA abundance is not different among the four strains (Figure 3F). Thus, the among-strain variation in vYFP signal must be due to a variation in translation. We also confirmed our results by a finer control of mCherry expression and ruled out the possibility that our observation is a byproduct of potential differences in translational accuracy among different *mCherry* versions (7; see Materials and Methods). Furthermore, because the accuracy hypothesis is based on *CAI* and thus predicts a higher vYFP expression in the strain carrying *mCherry*-4 than that carrying *mCherry*-3, our results (Figure 3D) are inexplicable by this hypothesis. Similarly, mechanisms resulting from translational errors, such as protein misfolding or aggregation, cannot explain our observation either.

In the experiment, we used *vYFP* to represent native genes in the yeast genome. However, because vYFP and mCherry have 71/220 = 32% of protein sequence identity, one might ask whether our observation can be generalized. Specifically, could the negative influence of mCherry expression on vYFP expression be caused entirely by the similarity in codon usage between *mCherry* and *vYFP*? We measured the codon usage dissimilarity between a pair of genes by a Euclidian distance and examined the distribution of this distance between each *mCherry* version and all yeast genes (Figure S8). The distribution is approximately bell shaped and the distance between *mCherry* and *vYFP* falls in the central part of the bell, suggesting that *mCherry* is no more similar to *vYFP* in overall codon usage than to average yeast genes. Furthermore, our results cannot be explained by amino acid similarity between mCherry and vYFP, because all *mCherry* versions have the same amino acid sequence and should not differentially affect vYFP expression through amino acid usage. Thus, our observation from *vYFP* can be extrapolated to native genes in the yeast genome.

### Why more highly expressed genes have stronger CUB

**Figure 4:**
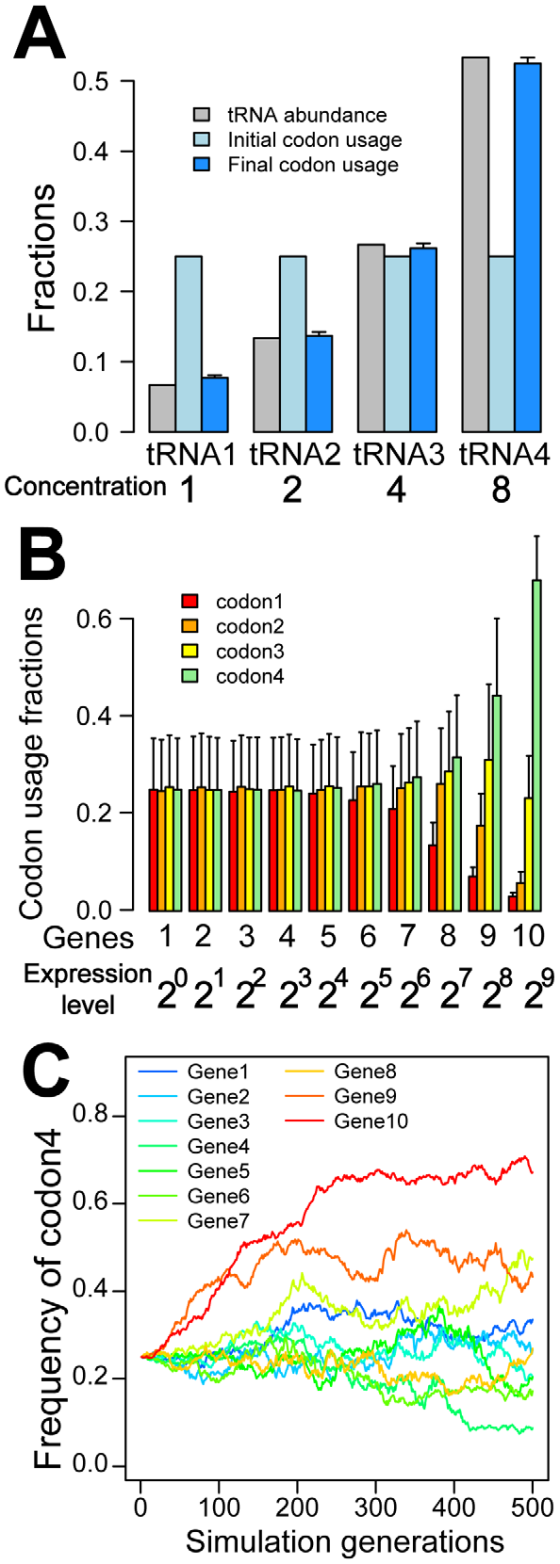
Computer simulation demonstrates that selection for translational efficiency results in the preferential use of codons with abundant cognate tRNAs in highly expressed genes. Ten genes with different expression levels are considered for a haploid organism. Four synonymous codons of an amino acid are each recognized by its cognate tRNA. Concentrations of the four tRNAs differ, but the initial codon frequencies are equal. Synonymous mutations, genetic drift, and natural selection for translational efficiency are considered (see Materials and Methods). (A) Overall changes of transcriptomic codon usage averaged from 1000 simulation replications. Error bars show one standard deviation. (B) Highly expressed genes evolved stronger codon usage biases than lowly expressed genes. The averages from 1000 simulation replications are presented. Error bars show one standard deviation. (C) Evolutionary changes in the usage of codon4, the codon recognized by the most abundant tRNA, in a randomly chosen simulation replication.

If translational efficiency is maximized when the cellular codon usage follows the proportional rule, why do highly expressed genes necessarily prefer codons with highly abundant cognate tRNAs and have stronger CUB than lowly expressed genes? We hypothesize that these phenomena are due to differential selective coefficients associated with synonymous mutations occurring in highly expressed and lowly expressed genes in the regain of the codon-tRNA balance upon a genetic perturbation. Let us imagine an amino acid with two synonymous codons (codon1 and codon2) that each uses a distinct tRNA species (tRNA1 and tRNA2) and assume that the present codon usage follows the proportional rule. Now, if the proportion of tRNA1 rises due to a mutation, natural selection will promote the fixations of synonymous mutations from codon2 to codon1 to reestablish the codon-tRNA balance. Such advantageous mutations occurring in highly expressed genes affect tRNA usage more than those occurring in lowly expressed genes and hence have a greater selective advantage and are fixed faster. This difference becomes even bigger when clonal interference [34] is considered. As a result, highly expressed genes use more codon1 and fewer codon2 than before and show stronger CUB. The contrasting scenario, in which the tRNA usage is rebalanced by frequent use of codon1 in lowly expressed genes, requires many synonymous substitutions in many lowly expressed genes, which will not happen because it takes much longer than rebalancing the tRNA usage by increasing codon1 frequency in highly expressed genes. Indeed, in a computer simulation of codon usage evolution that starts from the equal usage of 4 synonymous codons whose cognate tRNAs have different concentrations, the final usage of the codons, after 500 generations of random mutation, genetic drift, and natural selection for translational efficiency, follows the proportional rule (Figure 4A). More importantly, the preferential use of high-concentration tRNA species and strong CUB in highly expressed genes are seen from both the average of 1000 simulation replications (Figure 4B) and any one replication (Figure 4C). The standard deviations presented in Figure 4B indicate an extremely low probability for CUB to be stronger or a preferred codon to be used more frequently in lowly expressed genes than highly expressed genes. As expected, the phenomena in Figure 4 disappear when the natural selection for translational efficiency is removed in the simulation (Figure S9). These observations support our model that the high *CAI* of highly expressed genes is a byproduct of natural selection for an overall cellular efficiency in translation, rather than the direct product of stronger selection for translation efficiency in more highly expressed genes [6].

### Optimal amino acid usage under tRNA shortage

**Figure 5:**
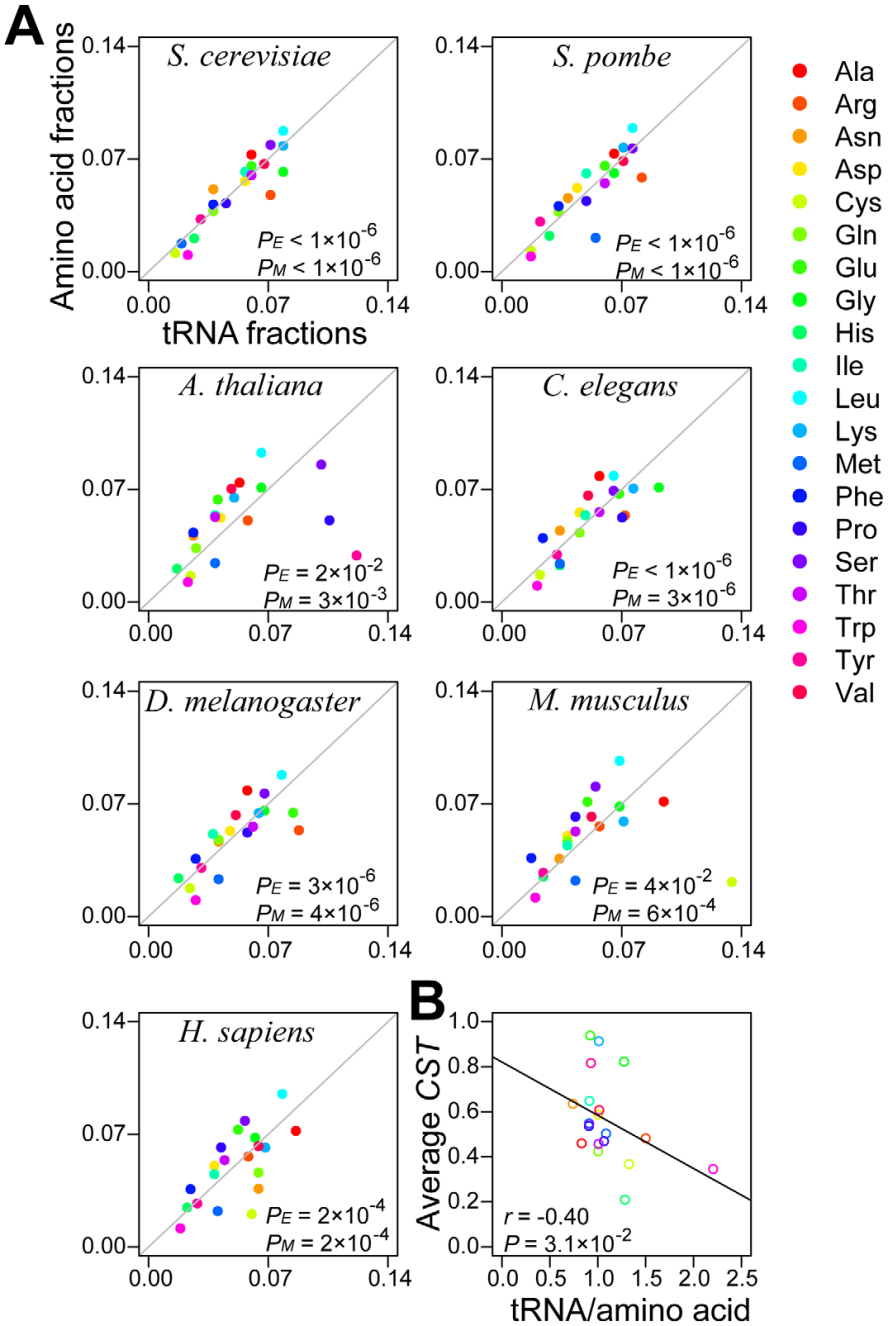
Amino acids are used approximately in proportion to cognate tRNA concentrations. (A) Relative uses of amino acids estimated from the transcriptomic data of 7 model eukaryotes are compared to the relative concentrations of their cognate tRNAs measured from gene copy numbers. The diagonal line shows the predicted proportional relationship between tRNA concentrations and cognate amino acid frequencies that maximizes translational efficiency under tRNA shortage. *P_E_* (or *P_M_*) is the probability that the Euclidian (or Manhattan) distance between the amino acid usage randomly generated under a uniform distribution and that predicted by the proportional rule is smaller than the observed distance, and is estimated from 10^6^ simulations. The distance definitions are the same as those in the legend of Figure 2, except that *i* is an amino acid instead of a codon. (B) The average *CST* of an amino acid in *S. cerevisiae* is negatively correlated with the availability of its cognate tRNAs, which is measured by the fraction of cognate tRNA genes among all tRNA genes divided by the frequency of the amino acid estimated from the transcriptome. The *P*-value is determined from 1000 permutations of *CST*s.

Analogous to synonymous codon usage, we predict that the optimal amino acid (or nonsynonymous codon) usage in speeding up translation is in proportion to the corresponding tRNA concentrations. Indeed, amino acid frequencies inferred from transcriptome data were reported to correlate positively with the corresponding tRNA gene copy numbers in yeast [35] and *C. elegans* [36]. More importantly, actual amino acid usage is significantly closer than random usage to our predicted optimal (i.e., the diagonal line in Figure 5A; *P*<10^−6^, simulation test). This phenomenon is also true in all other model eukaryotes examined, although the level of match between the observation and prediction varies among species (Figure 5A). Transcriptomic amino acid usages instead of proteomic amino acid usages are plotted here because the latter are unavailable for most species. Nevertheless, *S. cerevisiae* data showed an almost perfect correlation between transcriptomic and proteomic amino acid usages (Figure S10), indicating that the former is a good proxy for the latter. We also predict a positive correlation between aminoacyl tRNA synthetase concentration and corresponding tRNA concentration to enhance the efficiency of amino acid charging. Such a correlation is indeed found in *S. cerevisiae* (*r* = 0.45, *P* = 0.03; Figure S11).

If amino acid frequencies are in perfect proportion to tRNA concentrations, the mean *CST* for an amino acid should not vary among amino acids. This uniformity, however, is not observed in yeast (Figure S12), suggesting that amino acid usage is only roughly proportional to tRNA concentrations (Figure 5A), which may be due to mutational bias [37] or antagonistic selective pressures from factors such as physiochemical properties [38] and synthetic costs [39] of various amino acids. Our model predicts that the average *CST* of an amino acid increases with the decrease of the relative availability of tRNAs for the amino acid. Indeed, a negative correlation exists between the tRNA availability and *CST* for the 20 amino acids (Pearson's *r* = −0.40, *P* = 0.03, permutation test; Figure 5B). This finding reconfirms tRNA shortage in translation, explains in part why *CST*s of nonsynonymous codons vary, and indicates compromised translational efficiency due to other fitness effects of amino acid usage.

## Discussion

### The translational efficiency hypothesis of CUB

Results from several earlier experiments are consistent with the role of CUB in enhancing translational efficiency or reducing ribosome sequestering [12], [17]. For example, when expressing many synonymous versions of a green fluorescent protein (GFP) gene in *E. coli*, Kudla and colleagues reported that strains harboring high-*CAI GFP* genes tend to grow faster than those harboring low-*CAI GFP* genes, despite the lack of a correlation between the *GFP* protein expression level and its *CAI* [12]. Although these authors found no correlation between *CAI* and protein misfolding, their experiment was unlikely to be sensitive enough for quantifying GFP misfolding [12]. Thus, it could not rule out the possibility that the observed variation in fitness was entirely caused by CUB's influence on translational accuracy. By contrast, we were able to demonstrate CUB's impact on translational efficiency after excluding its impact on translational accuracy.

A recent study in *E. coli* showed that the ribosome shortage induced by over-expression of unneeded proteins can be alleviated by physiological adaptation in 30 to 40 generations, owing to the manufacture of additional ribosomes [40]. This finding suggests that the disadvantage of suboptimal codon usage may also be mitigated by physiological adaptation. Nevertheless, physiological adaptation takes time. If the growth rate fluctuates rapidly due to frequent environmental changes, the fitness of the individual with suboptimal codon usage is expected to be much lower than the individual with balanced codon usage.

We hypothesized and demonstrated that translational efficiency is optimized by codon-tRNA balance. This new model of translational efficiency by unequal codon usage differs substantially from the prevailing model (Table 1). One critical piece of evidence for our model is similar *CST*s of synonymous codons in wild-type yeast. Our *CST* estimation is based on the assumption that the time a codon occupies the ribosomal A site equals the waiting time for the cognate tRNA. Our estimates of all *CST*s would be biased upward to a similar level if downstream “traffic jams” happen during translational elongation. However, a recent study suggested that downstream traffic jams are unlikely, due to slow “ramps” at the beginning of an mRNA [21]. Furthermore, even if downstream traffic jams occur, it should affect synonymous codons as well as nonsynonymous codons and thus cannot explain why only synonymous codons but not nonsynonymous codons have similar *CST*s.

**Table 1:** Comparison between the old and new models of translational efficiency by unequal codon usage.

Comparisons	Old model	New model
Ternary complexes of aminoacylated tRNA+eEF-1á+GTP	In excess.	In shortage.
Translational speeds of synonymous codons in wild-type cells	Faster for those with higher cognate tRNA concentrations.	Equal, because codon usage has been optimized to be proportional to cognate tRNA concentrations.
Translational speeds of synonymous codons in mutant cells	Faster for those with higher cognate tRNA concentrations.	Unequal when the codon-tRNA balance is broken. Slower for codons with higher ratios between the codon fraction and the cognate tRNA fraction.
Why is the codon usage bias stronger in more highly expressed genes?	Fast translation of highly expressed genes is favored over fast translation of lowly expressed genes.	Synonymous mutations in highly expressed genes have larger effects than those in lowly expressed genes in regaining the codon-tRNA balance, which increases the overall translational efficiency of the cell.
Why is the codon usage proportional to cognate tRNA concentration?	No explanation.	It maximizes the overall cellular translational efficiency when ternary complexes are in shortage.
How to reach the highest cellular translational efficiency in making a synthetic cell?	Exclusive use of preferred codons.	Codon usage in proportion to cognate tRNA concentrations.

Over two decades ago, Curran and Yarus indirectly estimated relative *CST*s for 29 sense codons in *E. coli*, under the assumption that the probability of a frame shift in the translation of a codon is proportional to the *CST* of the codon [41]. They reported that only codons of very low *CST*s tend to be preferentially used [41]. However, because their fundamental assumption about the frame-shift rate is incorrect [42], their *CST* estimates are unlikely to be correct. It is also possible that prokaryotes and eukaryotes have some differences in using CUB to regulate translational efficiency (e.g., translational attenuation in prokaryotes). In another *E. coli* study, Sorensen and colleagues reported faster translation of a multicopy-plasmid-borne *lacZ* gene when a segment of the gene comprises mainly preferred codons than when it comprises mainly unpreferred codons [43]. This result cannot be used to infer relative *CST*s of synonymous codons in wild-type cells, because the extremely high expression of synonymous versions of the endogenous *lacZ* gene from plasmids potentially breaks the codon-tRNA balance and alters *CST*s. Nevertheless, their observation is fully compatible with our finding of different levels of translational efficiency induced by the expressions of different synonymous versions of *mCherry*. Several other studies reported similar findings [25], [44]. Recently, some authors calculated *CSTs* by assuming that the *CST* of a codon is determined by the relative concentrations of its cognate, nearly cognate, and non-cognate tRNAs without considering tRNA shortage or using ribosome profiling data [45]. Because of the violation of the fundamental assumption they made, their estimates are likely to be incorrect. Indeed, their estimated *CST*s would predict a slower translation of mCherry version 3 than 4, contradictory to our experimental result (Figure 3D). While the present work was under review, Ingolia and colleagues reported estimates of translational elongation speeds in mouse embryonic stem cells using a pulse-chase strategy that does not involve expressions of heterologous genes [46]. Although their method is different from ours, their finding of similar elongation speeds among synonymous codons is highly consistent with our results from yeast.

Our discoveries require reinterpretation of several earlier observations. For example, higher prevalence of codons with abundant cognate tRNAs in genes with higher expressions is often interpreted as a result of a stronger demand for fast translation of more abundant proteins [19]–[20]. This interpretation is not supported by our results. Rather, we suggested and demonstrated by simulation that, the selection coefficient for synonymous mutations that help achieve the codon-tRNA balance is greater in highly expressed genes than in lowly expressed genes, leading to quicker and more acquisitions of codons with abundant cognate tRNAs in the former than in the latter. In this regard, our results support that CUB serves as a global strategy to enhance the efficiency of the translation system [12], [47].

**Figure 6:**
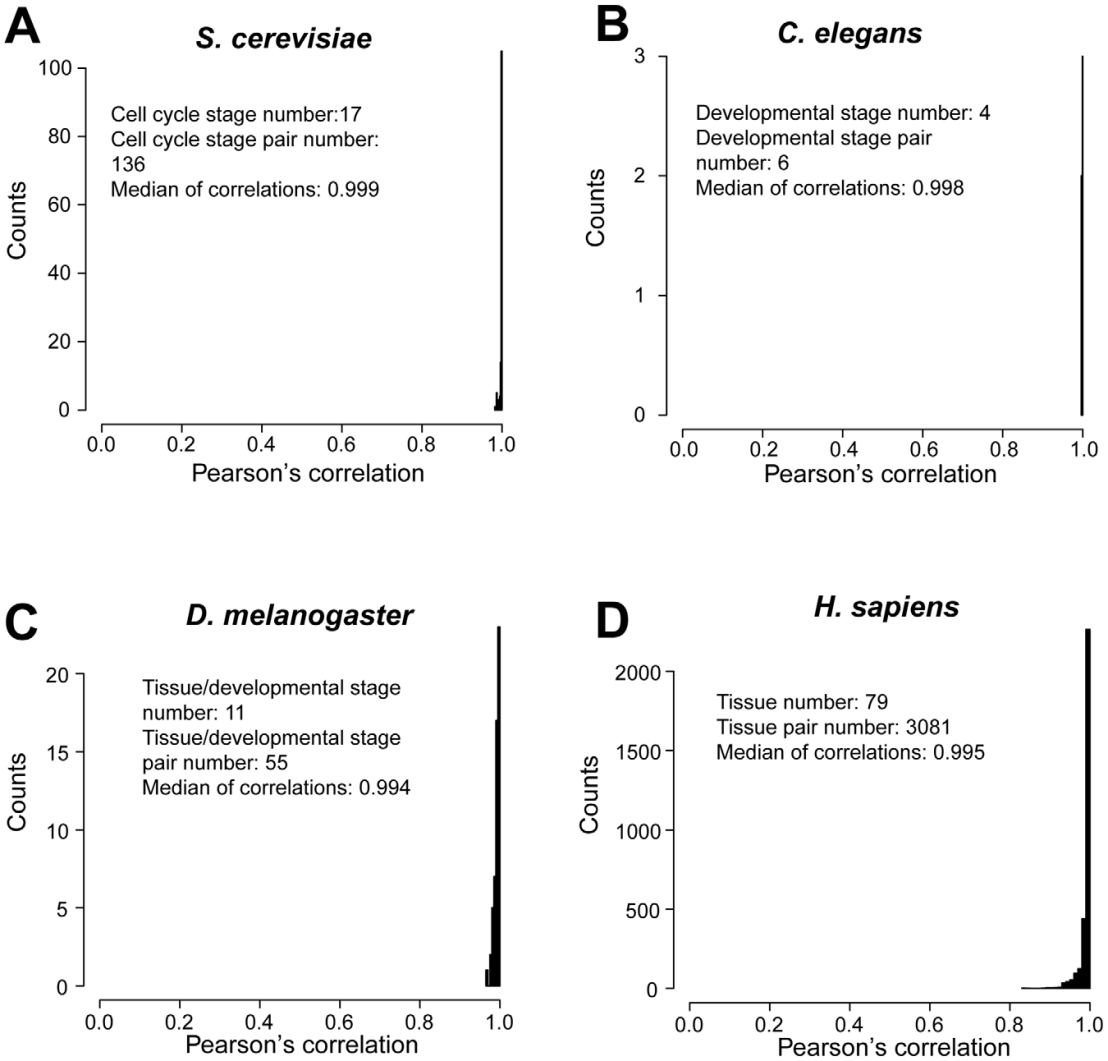
Similarity in transcriptomic codon usage across cell cycle stages, developmental stages, and tissues. Distributions of pairwise Pearson's correlations of codon usage among (A) mitotic cell cycle stages in *S. cerevisiae*, (B) developmental stages in *C. elegans*, (C) tissues and developmental stages in *D. melanogaster*, and (D) among tissues in *H. sapiens*.

Within an organism, the transcriptome can vary among cell cycle stages, developmental stages, and tissues. How do such variations affect the codon-tRNA balance? We found pairwise Pearson's correlations in transcriptomic usage of all 61 sense codons to be nearly 1 among different time points in the *S. cerevisiae* mitotic cell cycle (Figure 6). We further analyzed the transcriptomic usage of all 61 codons across tissues and/or developmental stages in the worm, fruitfly, and human. If multiple replications of the same cell type exist in a dataset, we randomly chose one replication in our analysis. Similarly high correlations were observed among different cell types within species (Figure 6). By contrast, the correlation is generally below 0.5 between any pair of the four species examined here. The high correlation in codon usage across cell cycle stages, developmental stages, and tissues of the same species is likely due to house-keeping genes, which are always highly expressed. Thus, within-organism gene expression variations have little impact on the maintenance of the codon-tRNA balance. Further, tRNA concentrations may covary with the transcriptomic codon usage to maintain the codon-tRNA balance across tissues [48].

A byproduct of our *CST* estimation is the translational initiation rate of each gene. We found that the translational initiation rate is significantly positively correlated with the mRNA concentration (ρ = 0.34, *P* = 6×10^−81^), suggesting a coordinated regulation of gene expression at the transcriptional and translational levels. We also observed a strong positive correlation between the translational initiation rate and *CAI* (ρ = 0.51, *P*<10^−196^), suggesting that *CAI* provides a moderate amount of information about the translational initiation rate. This may explain why the protein concentration correlates with the product of mRNA concentration and *CAI* better than with the mRNA concentration alone [49]. Several studies revealed reduced mRNA stability near the translation initiation site, suggesting that the reduced stability may enhance the translational initiation rate [12], [32]–[33]. Indeed, we found a weak but significant positive correlation between the reduction in mRNA stability [32] and our estimated translational initiation rate (ρ = 0.08, *P* = 1×10^−5^).

### Translational efficiency and accuracy are two separable benefits of CUB

Given that CUB improves both translational efficiency and accuracy, one wonders whether one of these effects is a side-effect of the other. For instance, it was previously suggested that the variation in translational accuracy among synonymous codons may be a byproduct of the variation in translational efficiency, because (i) most translational errors are believed to occur during codon selection, (ii) codon selection has been assumed to be faster for preferred codons than unpreferred codons, and (iii) faster codon selection is thought to result in fewer errors [50]. Because our result invalidates assumption (ii) for wild-type cells, the above argument no longer holds. Thus, even though translational accuracy may be affected by relative concentrations of tRNAs in engineered yeast cells with grossly imbalanced codon-tRNA usage [51], this impact is not expected in wild-type cells because our results strongly suggest that isoaccepting tRNA species have effectively the same concentrations in wild-type cells. In addition, the enrichment of preferred codons at evolutionarily conserved amino acid residues cannot be explained by the translational efficiency hypothesis [7]–[10]. Furthermore, experimental data showed that translational accuracies of iso-synonymous codons vary [52], suggesting that the variation in accuracy cannot be entirely caused by the variation in cognate tRNA concentration, because iso-synonymous codons use the same cognate tRNA. Rather, comparative genomic analyses strongly suggest that translational accuracy is likely to be intrinsically different among synonymous codons [1], [53]. Further, we were able to establish CUB's impact on translational efficiency even after we controlled its impact on translational accuracy (Figure 3, Figure S7). In addition, because translational accuracy is not entirely determined by translational efficiency [7]–[10], the proportional rule, which is predicted from selection for efficiency, is not predicted from selection for accuracy, especially because translational errors at different residues have different fitness effects. Thus, the impact on efficiency cannot be a byproduct of the impact on accuracy. Taken together, we conclude that translational accuracy and efficiency are two separable benefits of CUB.

### Evolutionary models of codon usage bias

**Figure 7:**
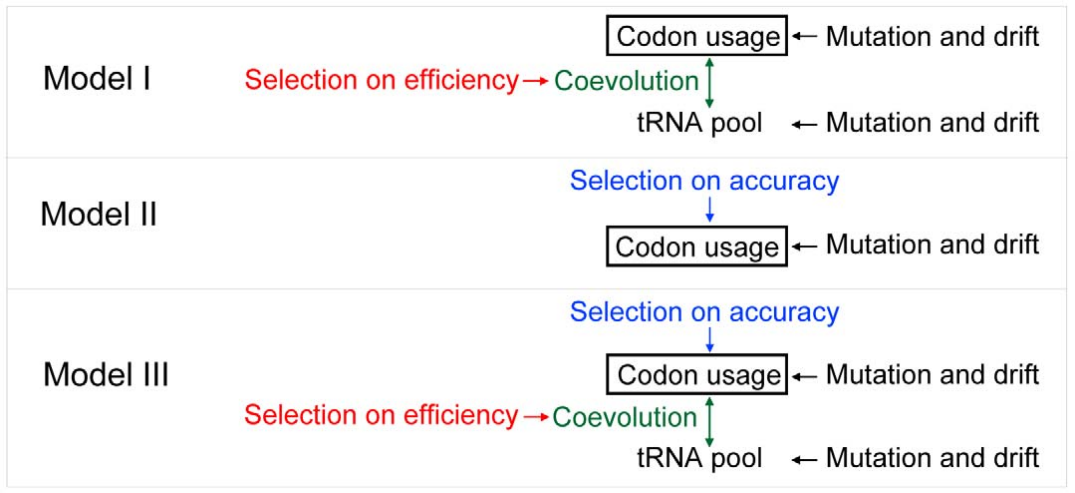
Evolutionary models of synonymous codon usage bias. Three models that differ in the involvement of natural selection for translational accuracy and efficiency in the evolution of codon usage bias. Models I and II can be rejected by the existing data, whereas model III is supported by available data.

Let us compare three evolutionary models of CUB that differ in the roles of translational accuracy and efficiency as the selecting agent. We also consider mutational bias and genetic drift, two known factors in the evolution of CUB, in these models. In model I, translational efficiency is the sole selecting force (Figure 7). This model predicts co-evolution of codon usage and cognate tRNA concentrations and a codon-tRNA balance at which the relative frequency of a synonymous codon (*p*_i_) equals the relative abundance of its cognate tRNA (*q*_i_). The expected values of *p*_i_ = *q*_i_ are determined by the mutational bias, which directly affects codon usage and indirectly affects tRNA concentrations. However, this model cannot explain the observation that, although preferred codons of an amino acid vary among species, this variation decreases substantially (but does not disappear) after the control of genomic GC content [1]. For example, GTT and GTA both code for valine and have the same GC content, but GTT is frequently used as the preferred codon when the genomic intergenic GC content is below 50% [1]. When the GC content exceeds 50%, GTG rather than GTC is often used as the preferred codon for valine [1]. This observation suggests that, in addition to translational efficiency, there is a separate selecting force with a relatively constant direction.

**Figure 8:**
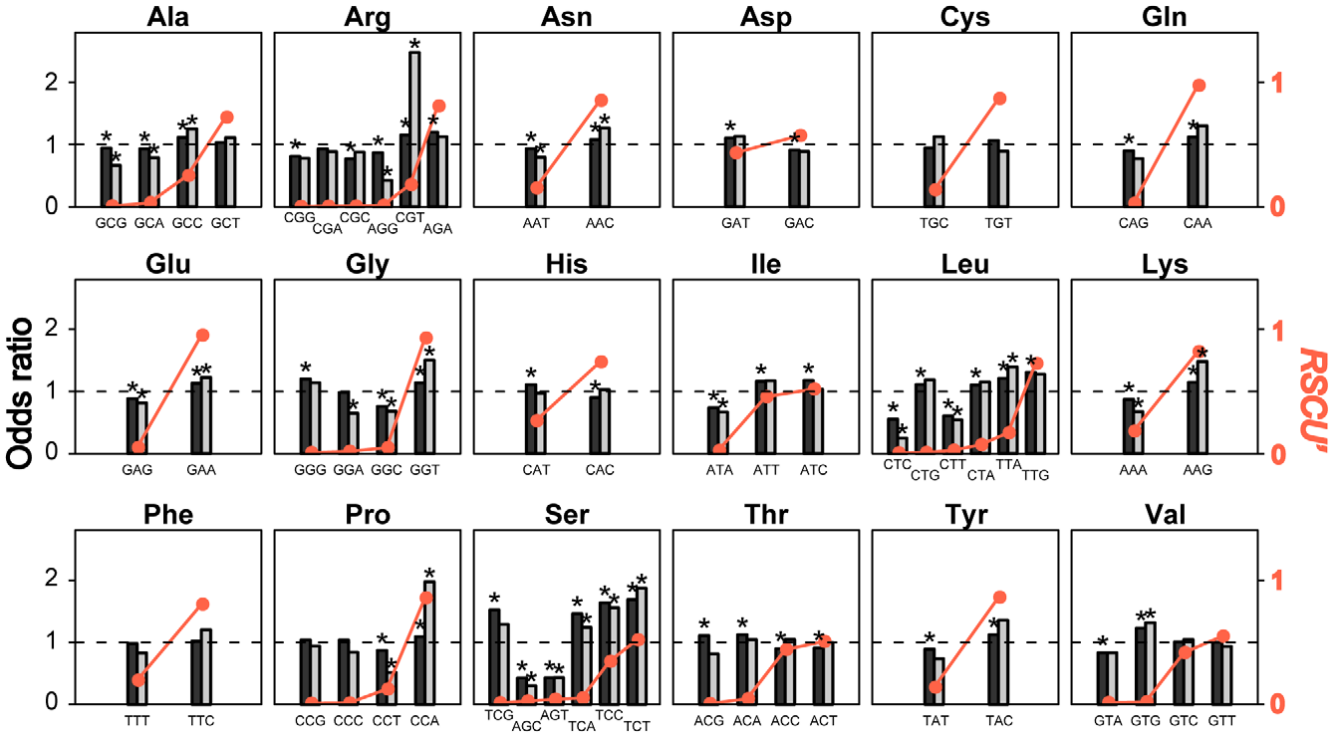
Matches and mismatches between preferred codons and accurate codons in *S. cerevisiae*. Odds ratio (bars) measures the enrichment of a synonymous codon at evolutionarily conserved amino acid residues relative to that at non-conserved residues and is used as a proxy for translational accuracy. *RSCU'* (orange dots) measures the preference in codon usage. Odds ratios are estimated from either all genes (black) or the 10% most highly expressed genes (grey) of *S. cerevisiae*. Asterisks indicate 5% significance in the deviation of an odds ratio from 1 (uncorrected for multiple testing).

In model II, translational accuracy is the sole selecting agent on CUB (Figure 7). The demand for translational accuracy, coupled with the mutational bias, determines the expected CUB, whereas selection for translational efficiency determines tRNA concentrations based on codon frequencies. The phenomenon of stronger CUB in more highly expressed genes is explainable by the protein-misfolding-avoidance hypothesis which predicts that highly expressed genes are translated more accurately by using accurate codons more frequently [7], [54]. Model II predicts that, after the control for the mutational bias, accurate codons are always the preferred codons in a species. If the translational accuracy of a codon is an intrinsic property of the codon and does not vary among species [29], we should observe no variation in the choice of preferred codons, after the control of mutational bias. This prediction, however, is incorrect, because preferred codons are not always the same in different species with the same mutational bias [1], [29]. A more rigorous test of this model is to compare the accurate and preferred codons of each amino acid in a species, because model II predicts a complete match between them. For each codon, we calculated an odds ratio by the relative use of the codon over other synonymous codons at conserved amino acid positions divided by that at non-conserved amino acid positions; the synonymous codon with the highest odds ratio is regarded as the most accurate codon because it is most preferentially used at important amino acid positions [7]–[10]. By comparing *S. cerevisiae* with its relative *S. bayanus*, we identified conserved and non-conserved amino acid positions. We calculated the odds ratio for each codon in each gene and then combined the odds ratios from all genes using the Mantel-Haenszel procedure [23]. By definition, the preferred codon of an amino acid is the one with the highest *RSCU'*. We found that, in 6 (Ala, Asp, Gly, His, Thr, and Val) of the 18 amino acids that have at least two synonymous codons, the codon with the highest odds ratio is different from the codon with the highest *RSCU'* (Figure 8). Furthermore, for three amino acids (Asp, His, and Thr), the codon with the highest *RSCU'* has an odds ratio significantly lower than 1 (Figure 8). We also used the 10% most highly expressed genes to calculate odd ratios; 8 (Ala, Arg, Asp, Cys, Ile, Leu, Thr, and Val) of the 18 amino acids show mismatches between the codon with the highest *RSCU'* and the codon with the highest odds ratio (Figure 8). These results provide unambiguous evidence for the inadequacy of model II.

In model III, selections for translational accuracy and efficiency jointly determine CUB (Figure 7). Let us consider three types of synonymous mutations with regard to their impacts on translational accuracy and efficiency. First, a synonymous mutation is likely to be fixed when it enhances both translational accuracy and efficiency, but is likely to be lost when it decreases both. Second, a synonymous mutation may increase the accuracy but reduce the efficiency. One possible outcome is that selection for higher accuracy will gradually alter the codon usage, which is followed by tRNA concentration changes that recover the loss of efficiency. Eventually, accurate codons will be the preferred codons. Alternatively, selection for higher accuracy may not be able to alter the codon usage permanently if the loss of efficiency is either too large or cannot be recovered by a corresponding tRNA change as quickly as the switch back of the codon usage. Consequently, accurate codons cannot become the preferred codons and the system is trapped in a local fitness peak that is the maximum for efficiency but not accuracy. For example, while codon CCA is more accurate than CCT for proline (Figure 8), there are still about a quarter of bacterial species with GC%<40 that use CCT as their preferred proline codon [1], suggesting that it is not rare for codon usage to be trapped in a local fitness peak. Third, a synonymous mutation may increase the efficiency but reduce the accuracy when the system is at a codon-tRNA imbalance. Although the fate of this mutation is determined by the relative strengths of the two forces, changes of tRNA concentrations could resolve the conflict better because they can increase efficiency without reducing accuracy. So, the final codon usage pattern will also depend on the rate of mutations that alter tRNA concentrations. While the quantitative aspects of model III require further exploration, it is clear that the model is able to explain, at least qualitatively, both the matches and mismatches between the accurate and preferred codons (Figure 8). It is also able to explain the codon-tRNA balance and the phenomenon of stronger CUB in genes with higher expressions. Thus, model III is most compatible with and best supported by available data. In addition to translational accuracy and efficiency, synonymous codon usage of individual genes may also be shaped by other forces, for example, those related to RNA splicing and stability [55]. But these forces are gene-specific and do not create genomic patterns of CUB.

### Implications for synthetic biology

Synthetic biology designs and constructs novel biological functions not found in nature. It has long been known that, in many but not all cases, increasing the *CAI* of a transgene boosts its protein expression [12], [56]–[57]. Different protein expression levels of synonymous transgenes are likely caused by *CST* differences created by various degrees of codon-tRNA imbalance induced by transgene expressions. Consistent with this idea, overexpression of rare tRNAs of *E. coli* (the bio-reactor) can rescue the tRNA depletion when heterologous human genes are expressed in *E. coli* [56]. When an artificially designed gene is added to a host cell, the potential imbalance between the overall cellular codon usage and the tRNA pool also affects the expressions of native genes and hence the growth of the host cell. We showed that *D*_ncu_, a newly devised index measuring the distance in codon usage between the transgene and the host cell, is an accurate indicator of the impact of per transgene protein molecule production on the expressions of native genes. We demonstrated that it is the *D*_ncu_ rather than *CAI* of the transgene that predicts its impact on the host protein expression. Therefore, *D*_ncu_ should be considered in synthetic biology when the impact of transgene expression on host gene expressions is a concern. Further, when genes from multiple species are assembled into a synthetic genome, designing tRNA gene numbers in proportion to the usage of their cognate codons will likely make protein expressions in the entire cell most efficient.

## Materials and Methods

### Yeast genomic data

The yeast ribosome profiling data [18] were downloaded from Gene Expression Omnibus (www.ncbi.nlm.nih.gov/geo/) under accession number GSE13750. Gene expression and protein expression levels were from http://web.wi.mit.edu/young/expression/ [58], http://www.imb-jena.de/tsb/yeast_proteome/ [59], and the supplementary data of a previous study [60]. Transcriptomic data for the yeast mitotic cell cycle were from a previous study [61]. Gene sequences and reading frames were downloaded from *Saccharomyces* Genome Database (SGD, www.yeastgenome.org). Numbers of tRNA gene copies were retrieved from an earlier study [22].

### Genomic data of other eukaryotes

Gene expression levels in *A. thaliana*, *D. melanogaster*, *M. musculus*, and *H. sapiens* were downloaded from Gene Expression Omnibus (GDS416, GDS2784, GDS592 and GDS596, respectively). Gene expression levels in *S. pombe* and *C. elegans* were retrieved from two earlier studies [62]–[63], respectively. Peptide and cDNA sequences of *S. pombe*, *A. thaliana*, *C. elegans*, *D. melanogaster*, *M. musculus*, and *H. sapiens* were from Ensembl (www.ensembl.org/). Numbers of tRNA gene copies in the above species were obtained from the genomic tRNA database (http://lowelab.ucsc.edu/GtRNAdb/).

### Estimation of codon selection time (*CST*)

Using the *S. cerevisiae* ribosome profiling data [18], we identified codons docked at the ribosomal A site, from the Illumina Genome Analyzer sequencing reads. By comparing the observed codon frequencies in the ribosome profiling data with the expected codon frequencies estimated from mRNA-Seq data generated under the same condition in the same experiment, we calculated the relative *CST*s of all 61 sense codons. Although Illumina sequencing may be biased toward certain sequences or nucleotides [64], this bias affects the mRNA-Seq and ribosome profiling data equally and thus will not affect our estimation of *CST*. For a sequencing read from the ribosome profiling data, nucleotide positions 16–18 were considered to be at the ribosomal A site where codon selection occurs [18]. Only those reads with exactly 28 nucleotides and 0 ambiguous sites were used to ensure the accurate determination of positions 16–18. We calculated the fraction of in-frame codons by comparing the read sequences with annotated yeast coding sequences. Consistent with what was previously reported [18], the majority of codons at positions 16–18 were in-frame in the ribosome profiling data. In the mRNA-Seq data, the fraction of each phase was close to one third, as expected. All out-of-frame codons were excluded. The probability of incorrect codon assignment was low, because only codons misaligned by at least 3 nucleotides may be assigned incorrectly. Transposons and uncharacterized genes were removed. Our *CST* estimation procedure (Figure S1) is as follows.

We first calculated *f_i_*, the observed frequency of codon *i*, in the ribosome profiling data by
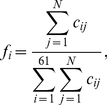
(1)where *c_ij_* is the count of codon *i* in mRNA *j* positioned at the ribosomal A site measured by ribosome profiling and *N* is the number of genes with ribosome profiling data (*N*>3000 for both rich and starvation conditions). The expected ribosome footprint frequencies of codon *i* (*F_i_*) when all codons have equal *CST* can be calculated based on the frequency of the codon in the mRNA-Seq data using
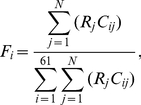
(2)where *R_j_* is the translational initiation rate of mRNA *j* and *C_ij_* is the count of codon *i* in mRNA *j* measured by mRNA-Seq. Then, the relative codon selection time for codon *i* is calculated by
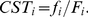
(3)We used an iterative approach to estimate the translational initiation rates that appear in Eq. 2. We first used *R_j_* = 1 for all *j*. After the *CST* is calculated for each codon, the elongation rate *e_j_* of mRNA *j* (i.e., the number of codons translated per unit time) is calculated by
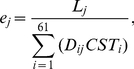
(4)where *L_j_* is the number of codons in each molecule of mRNA *j* and *D_ij_* is the number of codon *i* in each molecule of mRNA *j*. The translational initiation rate *R_j_* can be estimated from
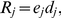
(5)where *d_j_* is the ribosome density on mRNA *j* (i.e., the number of ribosomes per codon) and can be estimated by
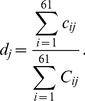
(6)We then used the newly estimated translational initiation rates to calculate *CST*s. After 10 iterations, *CST* estimates converge (Figure S2) and are considered as our final estimates. Because our estimates of *CST*s are relative values, we rescaled them by setting the maximal observed value at 1.

*CST* estimates from different experimental replicates were highly correlated (*r* = 0.79, *P* = 6×10^−14^) and were thus pooled for the rest of the analysis. Three different sets of initial values of translational initiation rates (uniform, proportional to *CAI* of each gene, inversely proportional to *CAI*) were used in *CST* estimation and they resulted in identical estimates of *CST*s (Figure S3A, S3B). Thus, *CST* estimation does not depend on the initial values of *R*. The standard errors of the *CST* estimates were estimated by bootstrapping genes present in the ribosomal profiling data 1000 times. The *CST* estimates from two different media (rich and starvation) are also very similar (Figure S3C). To ensure no mistake in the estimation of *CST*, the first two authors of this paper independently derived the formulas, wrote the computer programs, and estimated the *CST*s, and their results were virtually identical.

### Estimation of synonymous codon usage bias in yeast

There are two commonly used measures of synonymous codon usage bias. The first is the *relative synonymous codon usage* (*RSCU*), defined by the frequency of a codon relative to the average frequency of all of its synonymous codons in a set of highly expressed genes [19]. Codons with *RSCU*>1 are preferred and those with *RSCU*<1 are unpreferred. To compare the usage of all 61 sense codons, we also used *RSCU'* = *RSCU*/*n*, where *n* is the number of synonymous codons of an amino acid. *RSCU'* of a codon is the proportion of use of a given codon among synonymous choices in a set of highly expressed genes. The second commonly used measure of synonymous codon usage bias is the *codon adaptation index* (*CAI*), which is calculated for a gene, and measures its usage of high-*RSCU* codons [20]. Briefly, *CAI* of a gene is the geometric mean of *RSCU* divided by the highest possible geometric mean of *RSCU* given the same amino acid sequence. *CAI* is a positive number no greater than 1. The greater the *CAI*, the more prevalent are preferred codons in the gene.

We first selected 200 most highly expressed genes based on a previous study [59]. Sixteen of these genes did not have expression information in another study [58] and 4 had expression levels lower than 4 times the genomic average (2.7 mRNA/cell reported in an earlier study [58]). The remaining 180 highly expressed genes were used to calculate *RSCU* and *RSCU'* for each codon. Our *RSCU* estimates were highly correlated with those previously reported [20] (*r* = 0.995, *P*<0.001, permutation test). *CAI* was calculated for each yeast gene and for each version of *mCherry* based on the *RSCU* values obtained above, following a previous study [20].

We also estimated the effective number of codons (*Ncp*) for each gene, after controlling the GC content of the gene [65]–[66]. We separately estimated the frequency (*f*) of each of the 61 sense codons in each gene. We then estimated Spearman's rank correlation (ρ) between *Ncp* and *f* among all genes for each codon. Among synonymous codons, those with more negative ρ values are considered to be more preferred [1]. This dataset was used in Figure S4 only.

### Concentrations of ternary complexes in *E. coli*

It has been reported that the physiological concentration of the ternary complex is ∼200 nM for Phe tRNA and Lys tRNAs in *E. coli* [67]. Because the number of Phe tRNA and Lys tRNA molecules per cell is 1830 and 4300, respectively [68], we calculated that the Phe tRNA concentration is 1830/(6.02×10^23^)/(1.1×10^−15^) = 2.8×10^−6^ M = 2800 nM, where 6.02×10^23^ is the number of molecules per mole and 1.1×10^−15^ liter is the average volume of an *E. coli* cell. Similarly, Lys tRNA concentration is estimated to be 6500 nM. Thus, about 200/[(2800+6500)/2] = 4.3% of tRNAs are in ternary complexes. Because there are ∼1.2×10^4^ ribosomes per *E. coli* cell [68], ribosome concentration is ∼18,000 nM. Thus, the ratio in the concentration of ternary complexes to that of ribosomes is expected to be 200×20/18000 = 0.22, if Lys and Phe can represent all 20 amino acids in ternary complex concentration.

### Mathematical proof that proportional codon usage maximizes translational efficiency

Without loss of generality, we assume that an amino acid is encoded by synonymous codons 1 and 2, which are respectively recognized by isoaccepting tRNAs 1 and 2. Let the relative usage of the two codons be *p*_1_ and *p*_2_ = 1−*p*_1_ and the relative concentrations of the two tRNAs be *q*_1_ and *q*_2_ = 1−*q*_1_, respectively. Let the codon selection time for the two synonymous codons be *t*_1_ and *t*_2_, respectively. Thus, the expected codon selection time for the amino acid concerned is *t* = *p*_1_ *t*_1_+*p*_2_ *t*_2_. When tRNAs are in shortage, the local concentrations of tRNA 1 and 2 are *aq*_1_/*p*_1_ and *aq*_2_/*p*_2_, where *a* is a constant. Because codon selection time is proportional to the inverse of the local tRNA concentration, we have 
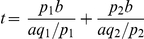
, where *b* is another constant. The above formula can be simplified to 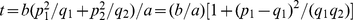. It is easy to find that *t* reaches its minimal value of *b*/*a* when 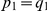 and 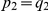. In other words, the expected codon selection time is minimized and thus translational efficiency is maximized when relative synonymous codon frequencies equal relative tRNA concentrations. Under this condition, codon selection time equals *b*/*a* for both codons and local tRNA concentration equals *a* for both tRNAs. A full treatment considering tRNA cycle and kinetics gave the same result [31].

### Empirical test of the proportional rule

We measured the Euclidian distance and Manhattan distance in synonymous codon usage from the observed values to the values predicted from the observed tRNA fractions using the proportional rule. To evaluate whether the observed distances are shorter than expected by chance, we conducted a computer simulation with 10^6^ replications under random codon usage. That is, the frequency of a synonymous codon is uniformly distributed between 0 and 1 with the constraint of the total frequency of all synonymous codons being 1. We then obtained the distribution of the distance between a random codon usage and the codon usage predicted from the observed tRNA fractions. We also conducted a second simulation with 10^6^ replications, in which tRNA factions vary randomly according to the above uniform distribution. We then obtained the distribution of the distance between the observed codon usage and that predicted from random tRNA fractions. This way, the potential confounding effect of genomic GC content on the assumed null distribution of codon usage becomes irrelevant to the test. We similarly tested the square rule and the truncation rule. Results from the first simulation are presented in Figure 2D, while those from the second simulation are in Table S1.

### Distance to native codon usage

We developed an index, distance to native codon usage (*D*_ncu_), to measure how different the codon usage of a (heterologous) gene is from the overall codon usage of the host cell, which is presumably balanced with tRNA concentrations. First, the Euclidean distance in synonymous codon usage between the heterologous gene and the host is calculated for each of the 18 amino acids with at least two synonymous codons by
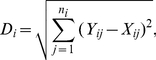
(7)where *Y_ij_* is the fraction of codon *j* among the synonymous codons of amino acid *i* for the heterologous gene and *X_ij_* is the fraction of codon *j* among the synonymous codons in the host transcriptome, *n_i_* is the number of synonymous codons for amino acid *i*. *D*_ncu_ of the gene is defined as the weighted geometric mean of *D_i_*, or
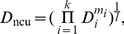
(8)where *k*≤18 is the number of amino acid types encoded by the gene excluding Met and Trp, which have no synonymous codons, *m_i_* is the number of amino acid *i* found in the protein, and *l* is the protein length excluding Met and Trp residues. By definition, *D*_ncu_ is between 0 and 1.

### Yeast experiments

The *mCherry* gene sequence was obtained from a previous study [69]. We designed four synonymous DNA sequences encoding the same mCherry peptide (Figure S5). The first 56 nucleotides were the same for all four sequences to avoid potential effects on the mRNA secondary structure, which affects protein translation [12], [32]–[33]. The GC contents of the four sequences (42–44%) were also made similar to each other and to the average value in yeast coding sequences (40%). In all sequences, synonymous codons were randomized in order and thus were unlikely to cause differences in order-related effects [27]. The different versions of *mCherry* DNA sequences were synthesized by Blue Heron Biotechnology. They were cloned into p426GPD [70] at SpeI and XhoI (New England Biolabs; Promega) and are under the control of the *GPD* promoter. The plasmids were subsequently transformed individually into a haploid yeast cell (BY4742) with *vYFP* [71] inserted into Chr XII [72]. The genotype of the cell is *MAT*α *his3*Δ*1 leu2*Δ*0 lys2*Δ*0 ura3*Δ*0 ho*Δ*0::P*_GPD_-*Venus*.

We measured the expressions of mCherry and vYFP in log growth phase in Yeast extract/Peptone/Dextrose (YPD) media by florescence-activated cell scanning (FACSCalibur, BD). Fluorescence of mCherry was measured from FL4 with a 670 nm pass filter and fluorescence of vYFP was measure from FL1 with a filter having a 30 nm bandpass centered on 530 nm. Yeast cells with mCherry fluorescence signals greater than the BY4742 negative control cells (i.e., mCherry fluorescence signals >10) were gated. We retrieved the forward scatter (FSC, which is proportional to cell size) and mCherry and vYFP fluorescence signals for all gated cells. The expression levels of fluorescent proteins were defined as their fluorescence signals divided by FSC. The mean mCherry expression level is 3.388±0.002, 6.468±0.007, 14.003±0.032, and 14.544±0.022 for the strains carrying *mCherry*-1, 2, 3, and 4, respectively. Expression levels of mCherry and vYFP were negatively correlated for each strain (*mCherry*-1: *r* = −0.22; *mCherry*-2: *r* = −0.57; *mCherry*-3: *r* = −0.60; *mCherry*-4: *r* = −0.62; *P*<2.2×10^−16^ in all cases). All gated cells were then grouped into 3 (Figure 3D) or 15 (Figure S7) bins with equal mCherry expression ranges. For each genotype, multiple independently transformed strains were examined on different days, but the results were highly similar. We thus combined all results obtained from different strains of the same genotype. The total numbers of cells measured were 456333, 648792, 352863, and 793832, respectively, for the strains carrying *mCherry*-1, 2, 3 and 4 (Figure 3B). To confirm that our results were not due to random secondary mutations, we removed the plasmids from each strain by using 5′-FOA media to select against the plasmids, and then measured the vYFP fluorescence intensities. We also sequenced the entire plasmid DNA from each of the four strains.

To compare the *vYFP* mRNA levels among strains, we extracted the total RNA (RiboPure-Yeast Kit, Ambion) from three independently transformed strains of each genotype. The total RNA was reversely transcribed into cDNA (Moloney Murine Leukemia Virus Reverse Transcriptase, Invitrogen) with random hexamer primers. The *vYFP* mRNA level was measured by quantitative polymerase chain reaction (7300 Real-Time PCR System, Applied Biosystems) with *ACT1* as an internal control. The primers for *vYFP* are 5′ – CATGGCCAACACTTGTCACT– 3′ and 5′ –TACATAACCTTCGGGCATGG– 3, while the primers for *ACT1* are 5′ - CTGCCGGTATTGACCAAACT - 3′ and 5′ – CGGTGATTTCCTTTTGCATT – 3′.

### Multivariate regression analysis

The software package RELAIMPO (http://cran.r-project.org/web/packages/relaimpo/) was used for a multivariate regression analysis of the yeast experimental data from all cells of the four strains. We compared the relative importance of *D*_ncu_ and *CAI* in explaining the among-cell variation in vYFP signal by the LMG method and used 1000 bootstrap replications to determine the statistical significance. Use of other methods (LAST, FIRST, and PRATT) implemented in RELAIMPO gave similar results.

### Impact of potential errors in translation on our experiments

Proponents of the translational accuracy hypothesis might argue that, because different synonymous codons have different mistranslation rates [52], [73] and preferred codons are considered to be more accurately translated than unpreferred codons [7], the *mCherry* with a low *CAI* is expected to produce fewer functional protein molecules than the *mCherry* with a high *CAI* even when the same numbers of protein molecules are produced. In other words, using red florescent signals may have led to a more severe underestimation of protein expression for the *mCherry* with a low *CAI* than for that with a high *CAI*. The average mistranslation rate has been estimated to be ∼5×10^−4^ per codon, and unpreferred codons have been posited to undergo mistranslation five times as often as preferred codons [7]. Based on these numbers and the *CAI*s of the four *mCherry* versions (Figure 3B), we assume that the mistranslation rate is 10×10^−4^, 8×10^−4^, 5×10^−4^, and 2×10^−4^ per codon for *mCherry*-1 to *mCherry*-4, respectively. Let us further assume that no mistranslated protein fluoresces. Given the length of mCherry (236 amino acids), we expect that 11.8%, 9.44%, 5.9%, and 2.36% of mCherry-1 to mCherry-4 proteins respectively fail to fluoresce due to mistranslation. On this assumption, we corrected mCherry expression levels from the observed florescent signals. We also conducted a better control of mCherry expression among strains by dividing cells of each strain into 15 bins based on the above corrected mCherry expression (Figure S7). Again, we observed a lower vYFP expression when the *D*_ncu_ of the *mCherry* gene is higher, across the range of mCherry expressions shared by the three strains (Figure S7). This result is conservative, because only a minority of mistranslations are expected to prevent fluorescence, and it is likely that we have overcorrected the effect of mistranslation.

### Computer simulation of the evolution of synonymous codon usage bias

We simulated the evolution of synonymous codon usage in an asexual haploid unicellular digital organism. In this organism, we focused on a single amino acid with four synonymous codons (codon1 to codon4) that are respectively recognized by four distinct tRNA species (tRNA1 to tRNA4). We assume that the relative concentrations of the four tRNA species are 2^0^, 2^1^, 2^2^, and 2^3^, respectively. The digital organism has ten genes with relative (mRNA and protein) expression levels from 2^0^ to 2^9^, respectively. These genes each have 12 codons that are sampled from the four synonymous codons. We started the simulation with exactly the same usage of the four synonymous codons in each gene. Synonymous mutations among codons all have the same rates and the total mutation rate per genome is assumed to be one synonymous change per generation. The relative *CST* for a codon is assumed to equal the number of times the codon is used in translation divided by the number of corresponding tRNA molecules. The total time (*T*) required for translating all the proteins can be considered as the generation time. *T* can be calculated by summing up the *CSTs* of all codons in all transcripts if there is only one ribosome in the cell. If there are *m* ribosomes in the cell, the time required would simply be *m* times shorter. Thus, without loss of generality, we assume *m* = 1. A strain with a shorter generation has a higher fitness and will spread in the population. Genetic drift is simulated by random sampling of cells for the next generation. The population size is 10^4^ individuals and the simulation lasts for 500 generations. We repeated the simulation 1000 times. Our results did not change when we simulated the evolution for more generations. By contrast, when we removed the natural selection for translational efficiency in simulation, the phenomena observed in Figure 4 disappeared (Figure S9).

Note that, in the simulation, we allow codon usage to evolve while fixing tRNA concentrations. If tRNA concentrations evolve while the codon usage is fixed, we also expect to observe the rebalance of codon-tRNA usage, but the correlation (or the lack of) between CUB and gene expression level will not change during this evolutionary process. In reality, tRNA concentrations and synonymous codon usage likely co-evolve to regain the balance. As long as codon usage is allowed to evolve, we expect stronger CUB to appear in more highly expressed genes, as demonstrated in Figure 4.

## Supporting Information

Figure S1 The procedure for estimating codon selection times (*CST*s) from ribosome profiling data. Circled numbers correspond to the equations in Materials and Methods and thick arrows show the iterations.
(PDF)

Figure S2 The estimates of *CST*s quickly converge after a few iterations.
(PDF)

Figure S3 Robustness of *CST* estimates. (A–B) Comparison of *CST* estimates when different initial values of translational initiation rates are used. (C) *CST* estimates from two media (rich and starvation) are similar.
(PDF)

Figure S4 No correlation between codon preference (red dots) and *CST* (grey bars) among synonymous codons. *CST*s are rescaled such that the maximal observed value is 1. Error bars show one standard error, estimated by the bootstrap method. Following ref. 1 in the main text, we measured the preference of a codon by Spearman's rank correlation (ρ) between the frequency of the codon in a gene and the effective number of codons in the gene (*Ncp*) across all genes (see Supplementary Methods). Preferred codons have more negative ρ values.
(PDF)

Figure S5 Alignment of the DNA sequences of the four synonymous versions of *mCherry* used in our yeast experiments. Invariant sites among the four sequences are marked with asterisks.
(PDF)

Figure S6 Codon usage of four synonymous versions of *mCherry* and that of the native transcriptome, compared to relative concentrations of cognate tRNAs in *S. cerevisiae*, for the 12 amino acids that have at least two isoaccepting tRNA species.
(PDF)

Figure S7 The impact of synonymous codon usage of *mCherry* on vYFP expression is not explainable by the translational accuracy hypothesis. The mCherry expression levels have been corrected by considering mistranslations that reduce the red florescent signals of mCherry. Mistranslation rates are assumed to be 10×10^−4^, 8×10^−4^, 5×10^−4^ and 2×10^−4^ per codon for *mCherry*-1 to *mCherry*-4, respectively. Our results are not sensitive to these assumptions of mistranslation rates. Cells of each strain are then divided into 15 equal-size bins by the corrected mCherry expression level per unit cell size. Error bars show one standard error.
(PDF)

Figure S8Distribution of the Euclidian distance in codon usage between all yeast genes and (A) *mCherry*-1, (B) *mCherry*-2, (C) *mCherry*-3, and (D) *mCherry*-4. Euclidian distance is calculated by 
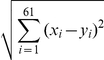
, where *x_i_* is the frequency of codon *i* in *mCherry* and *y_i_* is the corresponding frequency in a yeast gene. The distance between *vYFP* and *mCherry* is indicated by the arrow.(PDF)

Figure S9 Results from computer simulations without selection for translational efficiency. The simulations are conducted as described in Materials and Methods, except that no selection for translational efficiency is applied. (A) Overall changes of transcriptomic codon usage averaged from 1000 simulation replications. Error bars show one standard deviation. (B) No significant difference in codon usage among genes of different expression levels. The averages from 1000 simulation replications are presented. Error bars show one standard deviation.
(PDF)

Figure S10 High correlation between amino acid frequencies inferred from yeast transcriptomic data and those from yeast proteomic data. Each dot represents an amino acid.
(PDF)

Figure S11 Correlation between the total tRNA gene copy number for an amino acid and the mRNA expression level of the corresponding aminoacyl tRNA synthetase. Each dot represents an amino acid. Only 18 amino acids are presented because of the lack of information for the synthetases of Pro and Glu. The aminoacyl tRNA synthetase genes were identified based on gene annotations in SGD (http://www.yeastgenome.org/) and the expression levels of these genes were obtained from Holstege et al. (1998 Cell 95, 717).
(PDF)

Figure S12Significantly different *CST*s among different amino acids. To quantify potential variations in *CST* among amino acids and among synonymous codons, we linearly regressed the *CST*s of the 61 sense codons using the formula of 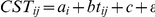, where *CST_ij_* is the *CST* of the *j*th codon of the *i*th amino acid, *a_i_* is the effect of amino acid *i*, *b* is the coefficient for the tRNA effect, *t_ij_* is the gene copy number for the cognate tRNA of the *j*th codon of the *i*th amino acid, *c* is a constant equal to the mean *CST* of all sense codons, and *ε* is the residual effect. The parameters in the above linear regression were estimated by the least squares method. Asterisks indicate a statistically significant effect (*, nominal *P*<5%; **, nominal *P*<1%). Note the lack of a significant effect of the cognate tRNA gene copy number on the variation of synonymous *CST*s, consistent with the results in Figure 1.(PDF)

Table S1 Euclidian and Manhattan distances between the observed tRNA fractions and the predictions of the proportional rule, square rule, and truncation rule, respectively.
(PDF)

Table S2 Unequal use of iso-synonymous codons in *S. cerevisiae*.
(PDF)
