# Supplementary figures and images for: Balanced Codon Usage Optimizes Eukaryotic Translational Efficiency
Source: PLoS Genet. 2012 Mar 29;8(3):e1002603. doi: 10.1371/journal.pgen.1002603 (PMC3315465; doi:10.1371/journal.pgen.1002603)

CST

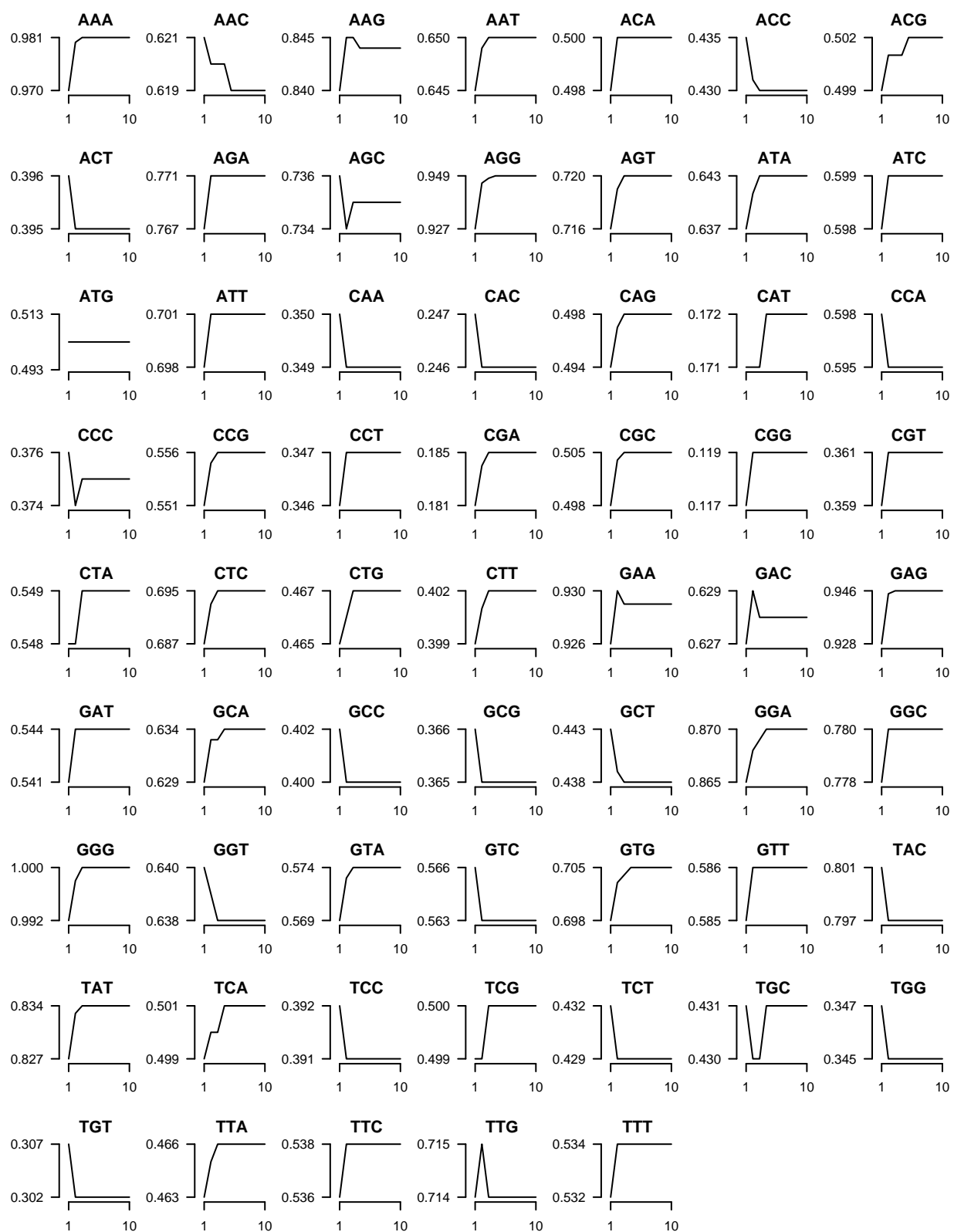

Iterations

Figure S2

Supplement: Figure S2 — The estimates of CSTs quickly converge after a few iterations. (PDF) [file pgen.1002603.s002.pdf]

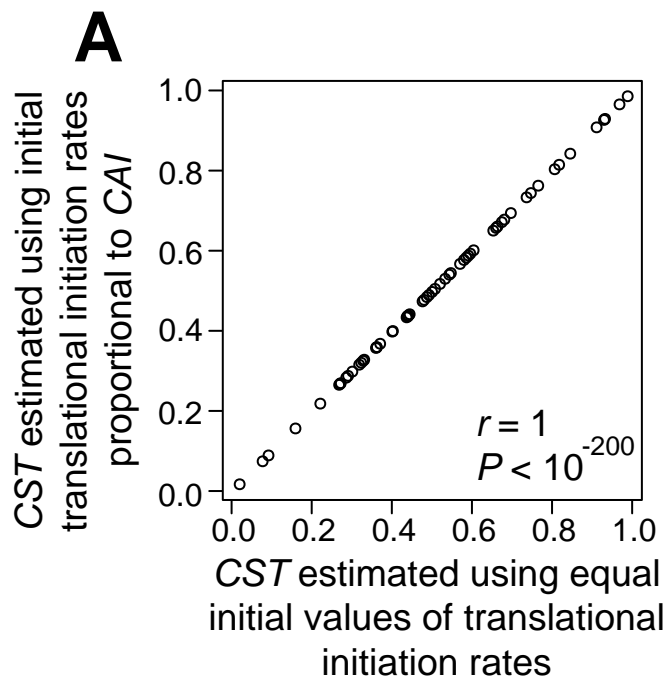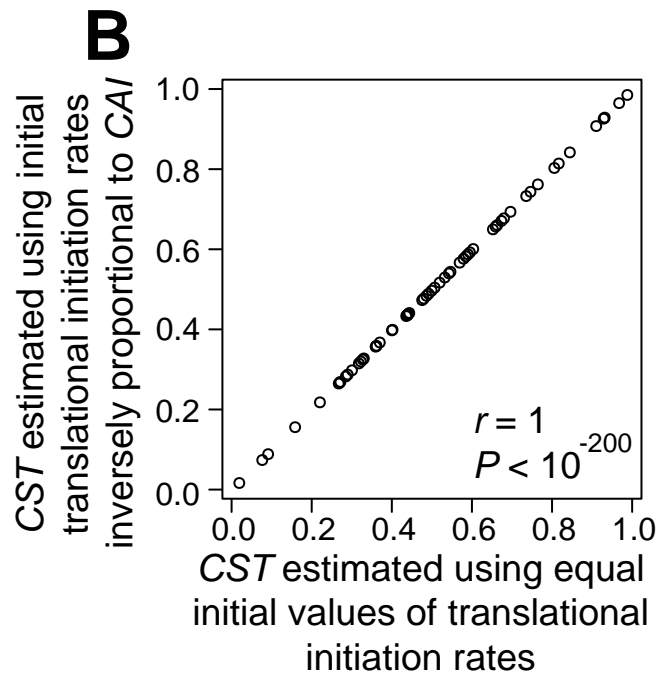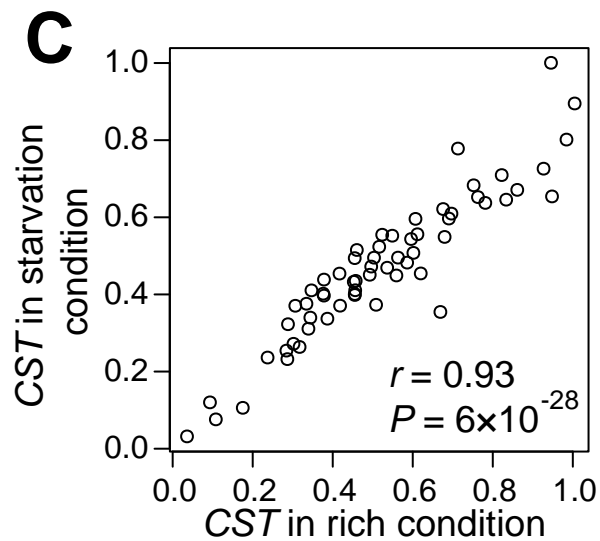

Figure S3

Supplement: Figure S3 — Robustness of CST estimates. (A–B) Comparison of CST estimates when different initial values of translational initiation rates are used. (C) CST estimates from two media (rich and starvation) are similar. (PDF) [file pgen.1002603.s003.pdf]

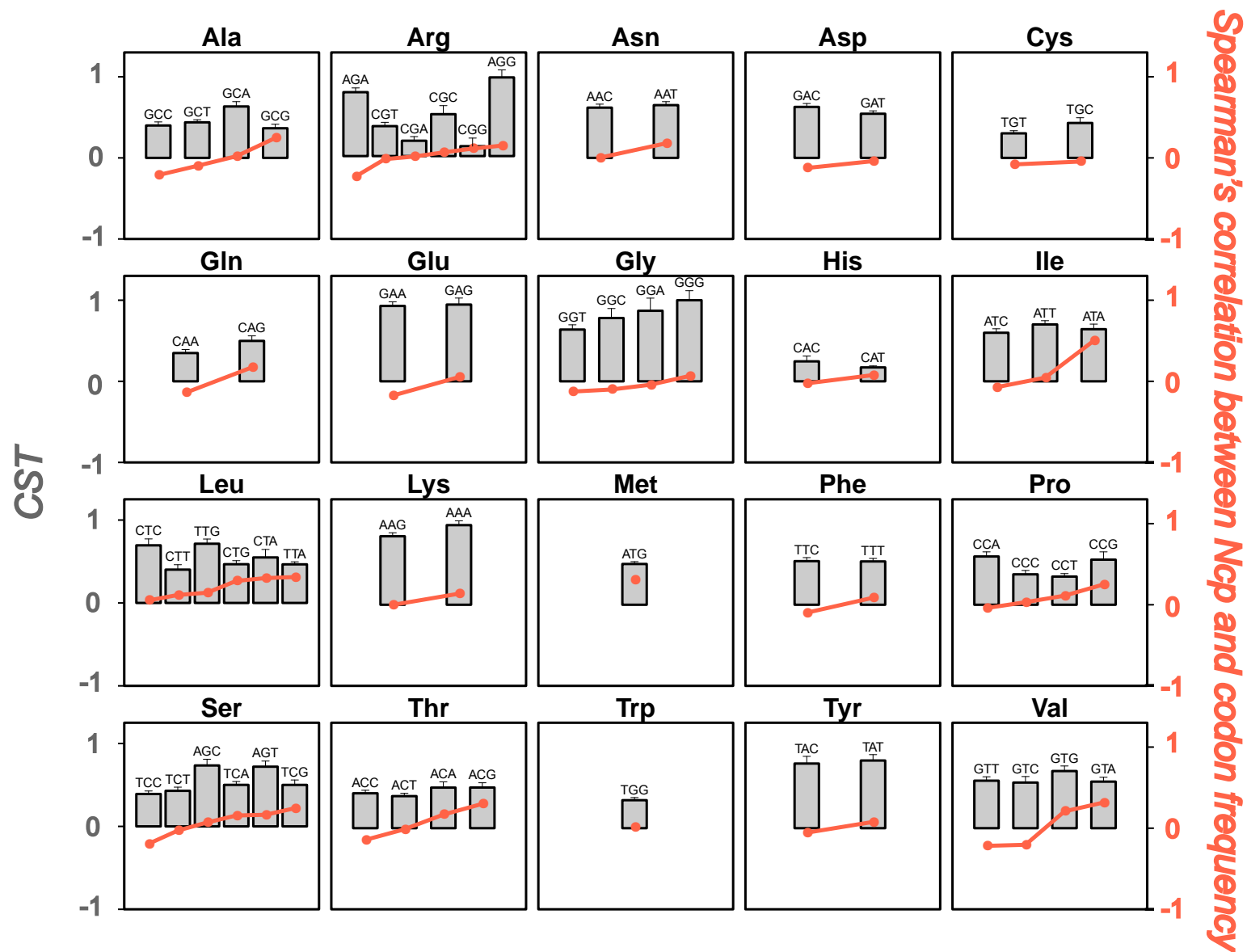

Figure S4

Supplement: Figure S4 — No correlation between codon preference (red dots) and CST (grey bars) among synonymous codons. CSTs are rescaled such that the maximal observed value is 1. Error bars show one standard error, estimated by the bootstrap method. Following ref. 1 in the main text, we measured the preference of a codon by Spearman's rank correlation (ρ) between the frequency of the codon in a gene and the effective number of codons in the gene (Ncp) across all genes (see Supplementary Methods). Preferred codons have more negative ρ values. (PDF) [file pgen.1002603.s004.pdf]

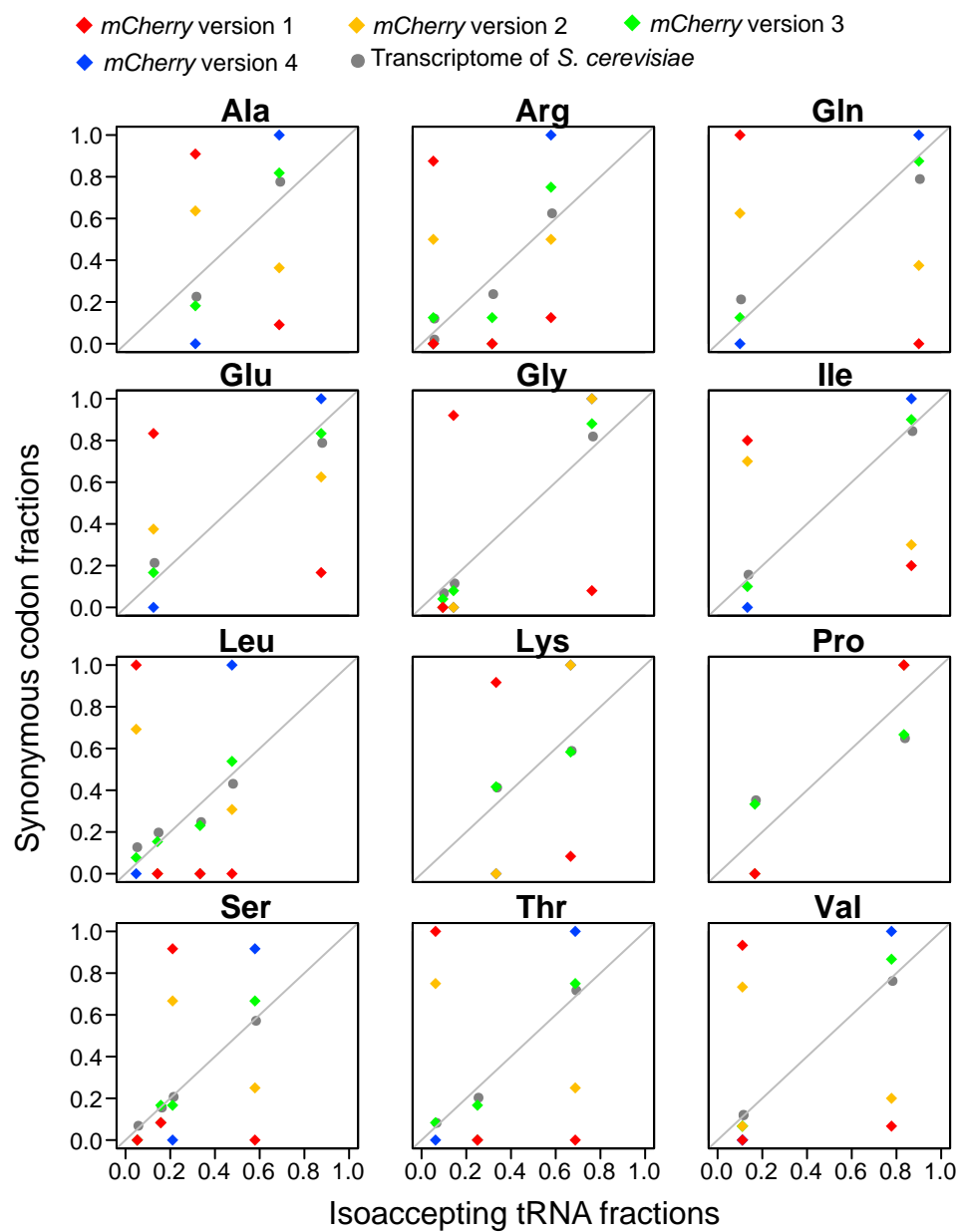

Figure S6

Supplement: Figure S6 — Codon usage of four synonymous versions of mCherry and that of the native transcriptome, compared to relative concentrations of cognate tRNAs in S. cerevisiae, for the 12 amino acids that have at least two isoaccepting tRNA species. (PDF) [file pgen.1002603.s006.pdf]

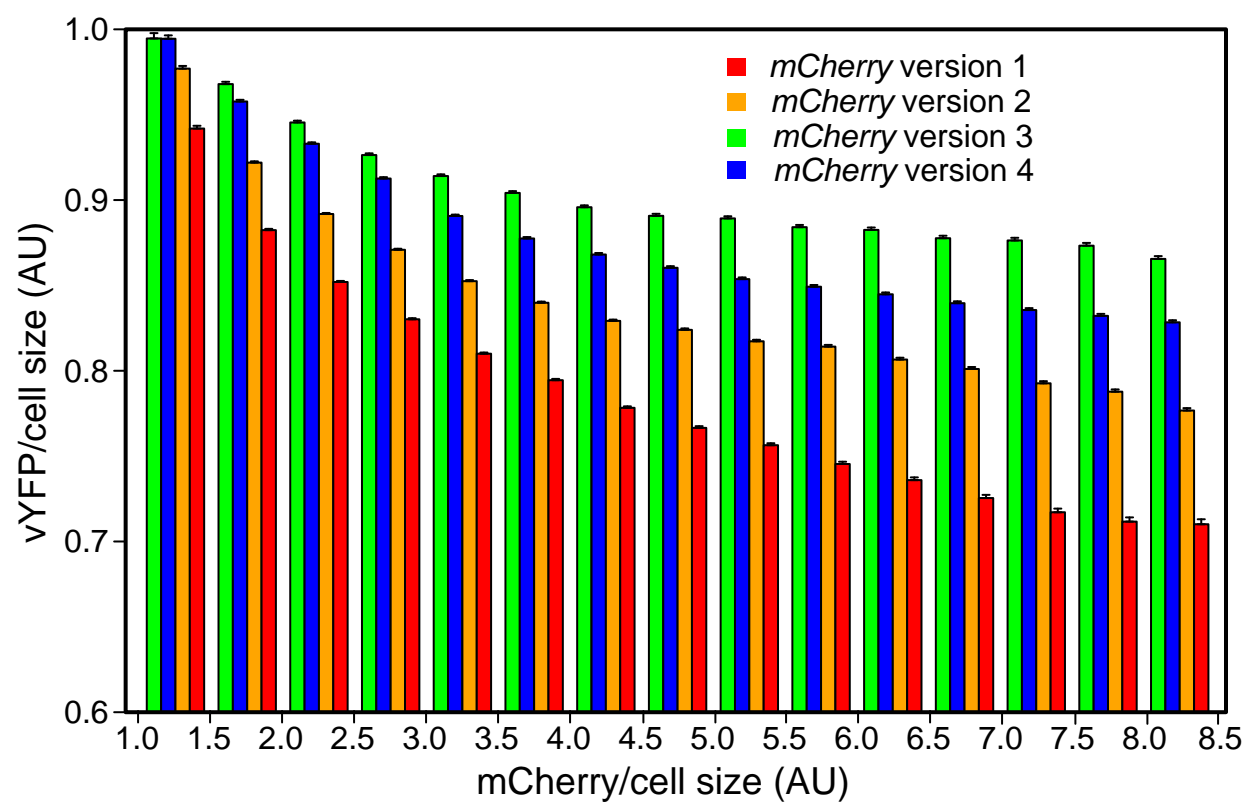

Figure S7

Supplement: Figure S7 — The impact of synonymous codon usage of mCherry on vYFP expression is not explainable by the translational accuracy hypothesis. The mCherry expression levels have been corrected by considering mistranslations that reduce the red florescent signals of mCherry. Mistranslation rates are assumed to be 10×10−4, 8×10^−4^, 5×10^−4^ and 2×10^−4^ per codon for mCherry-1 to mCherry-4, respectively. Our results are not sensitive to these assumptions of mistranslation rates. Cells of each strain are then divided into 15 equal-size bins by the corrected mCherry expression level per unit cell size. Error bars show one standard error. (PDF) [file pgen.1002603.s007.pdf]

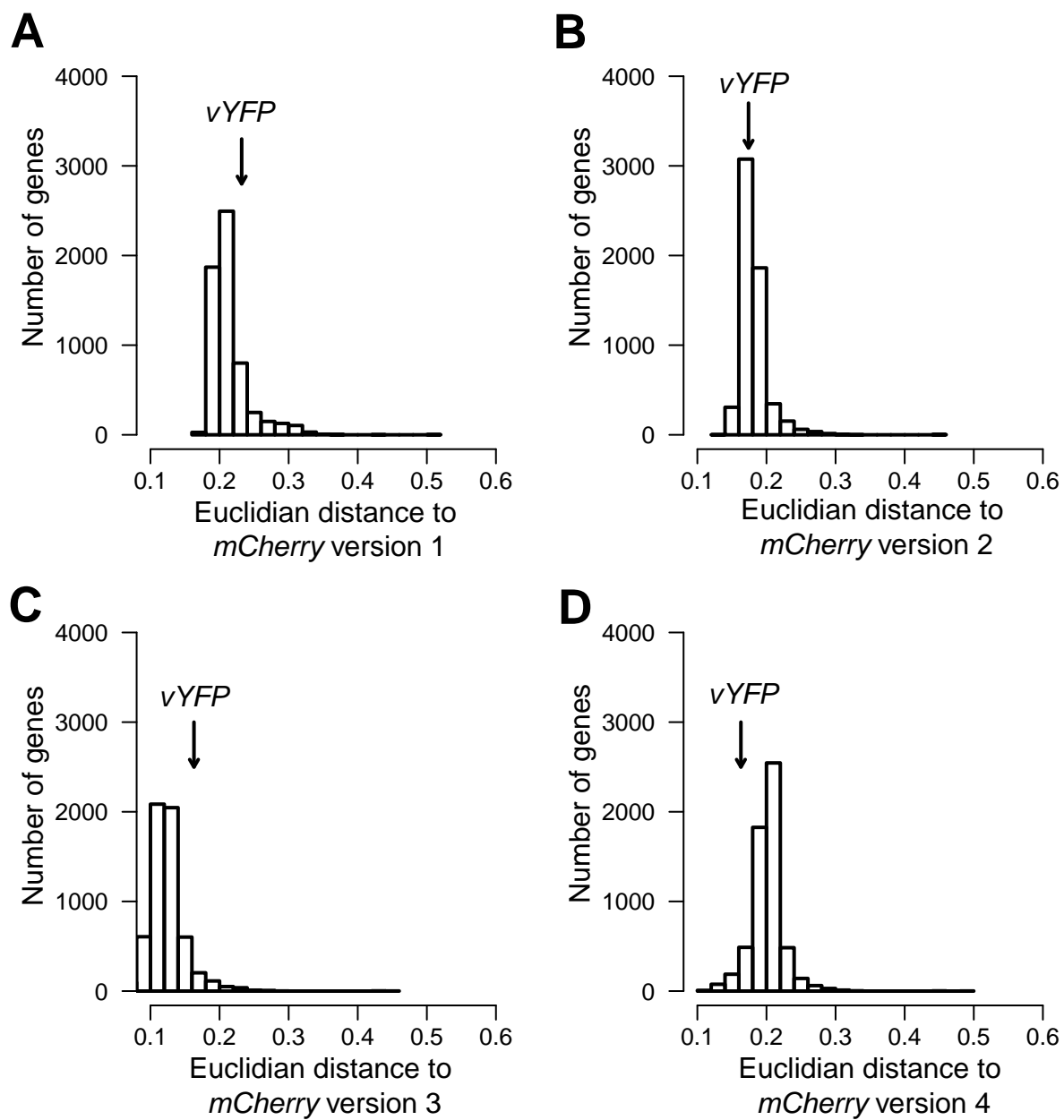

Figure S8

Supplement: Figure S8 — Distribution of the Euclidian distance in codon usage between all yeast genes and (A) mCherry-1, (B) mCherry-2, (C) mCherry-3, and (D) mCherry-4. Euclidian distance is calculated by , where xi is the frequency of codon i in mCherry and yi is the corresponding frequency in a yeast gene. The distance between vYFP and mCherry is indicated by the arrow. (PDF) [file pgen.1002603.s008.pdf]

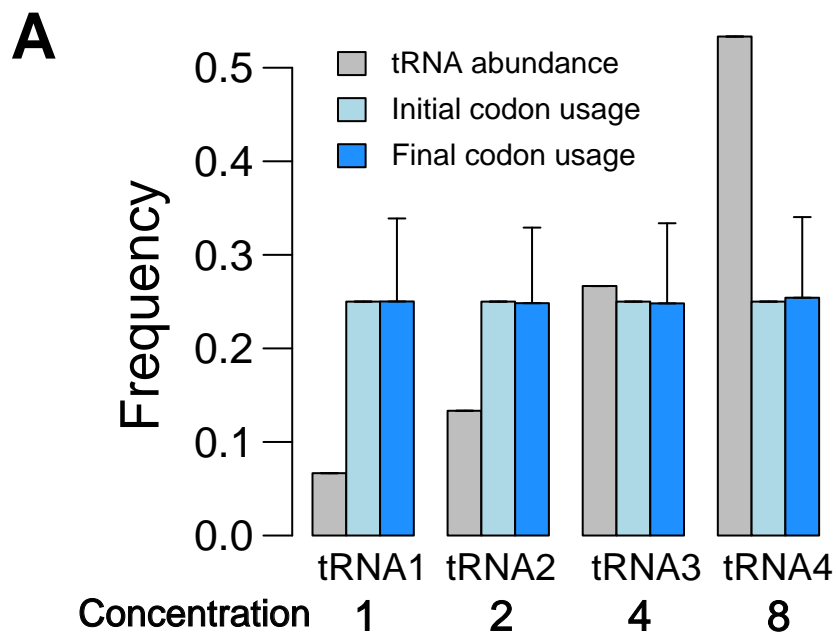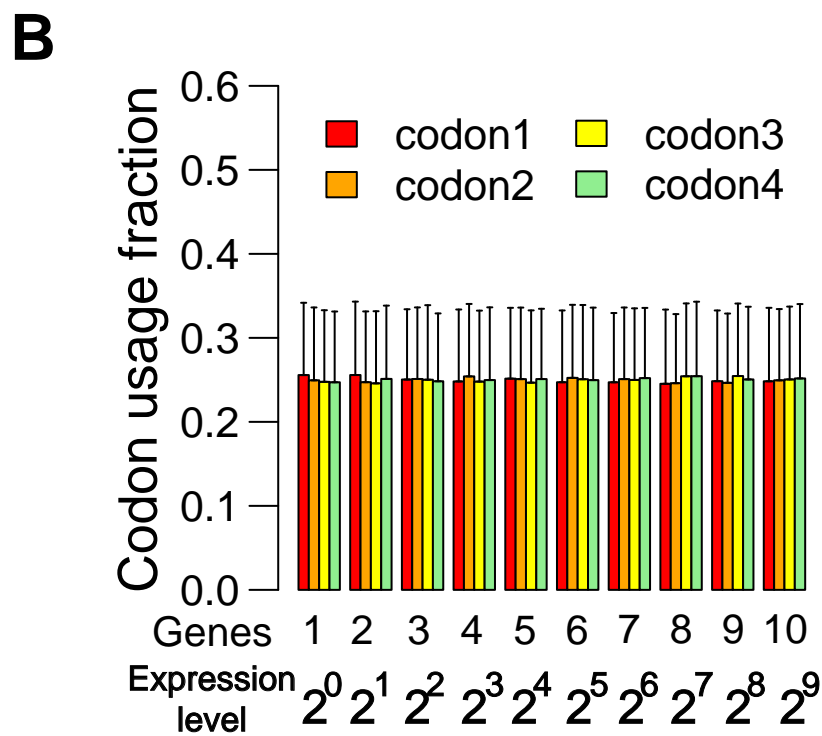

Figure S9

Supplement: Figure S9 — Results from computer simulations without selection for translational efficiency. The simulations are conducted as described in Materials and Methods, except that no selection for translational efficiency is applied. (A) Overall changes of transcriptomic codon usage averaged from 1000 simulation replications. Error bars show one standard deviation. (B) No significant difference in codon usage among genes of different expression levels. The averages from 1000 simulation replications are presented. Error bars show one standard deviation. (PDF) [file pgen.1002603.s009.pdf]

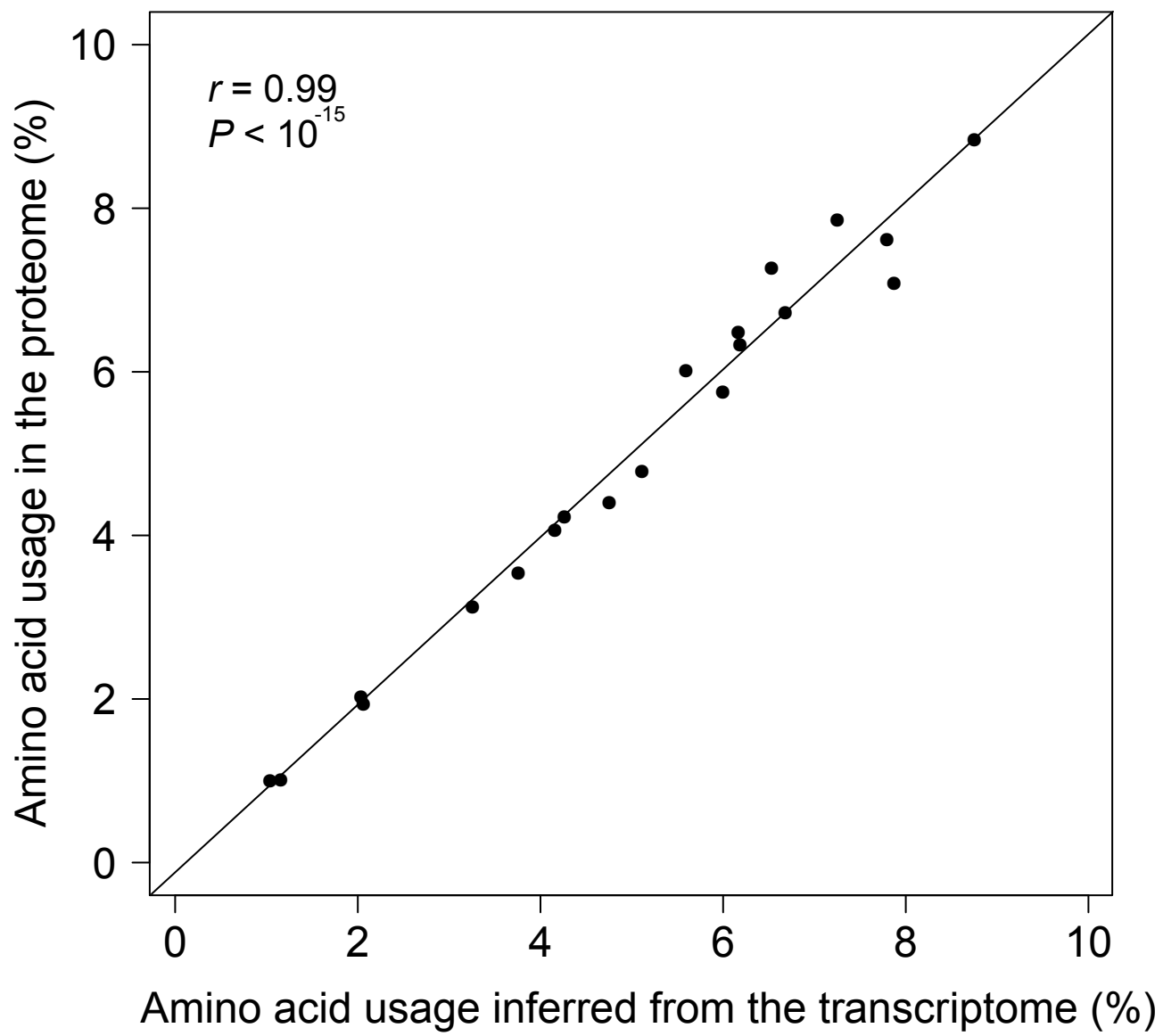

Figure S10

Supplement: Figure S10 — High correlation between amino acid frequencies inferred from yeast transcriptomic data and those from yeast proteomic data. Each dot represents an amino acid. (PDF) [file pgen.1002603.s010.pdf]

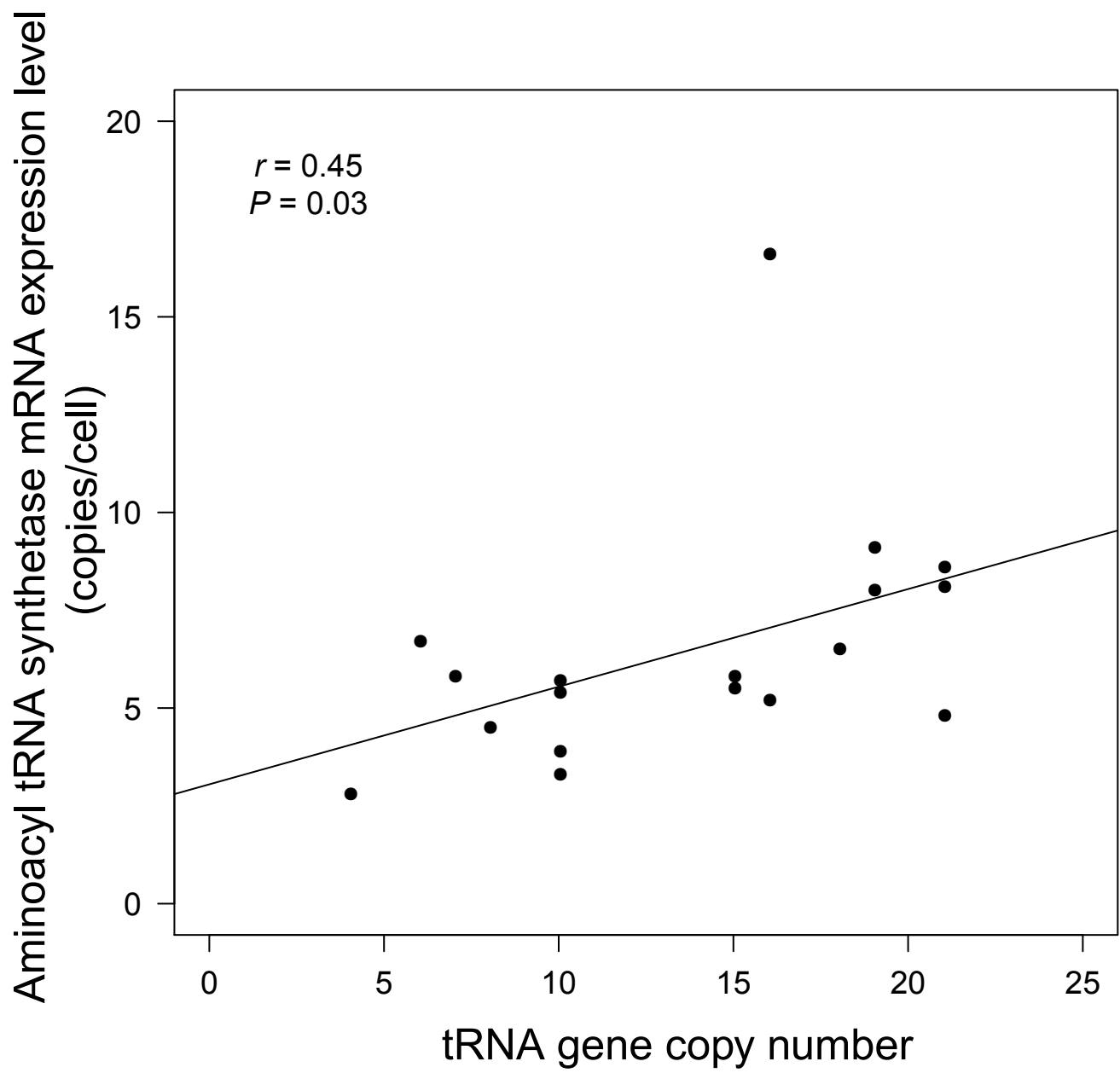

Figure S11

Supplement: Figure S11 — Correlation between the total tRNA gene copy number for an amino acid and the mRNA expression level of the corresponding aminoacyl tRNA synthetase. Each dot represents an amino acid. Only 18 amino acids are presented because of the lack of information for the synthetases of Pro and Glu. The aminoacyl tRNA synthetase genes were identified based on gene annotations in SGD (http://www.yeastgenome.org/) and the expression levels of these genes were obtained from Holstege et al. (1998 Cell 95, 717). (PDF) [file pgen.1002603.s011.pdf]

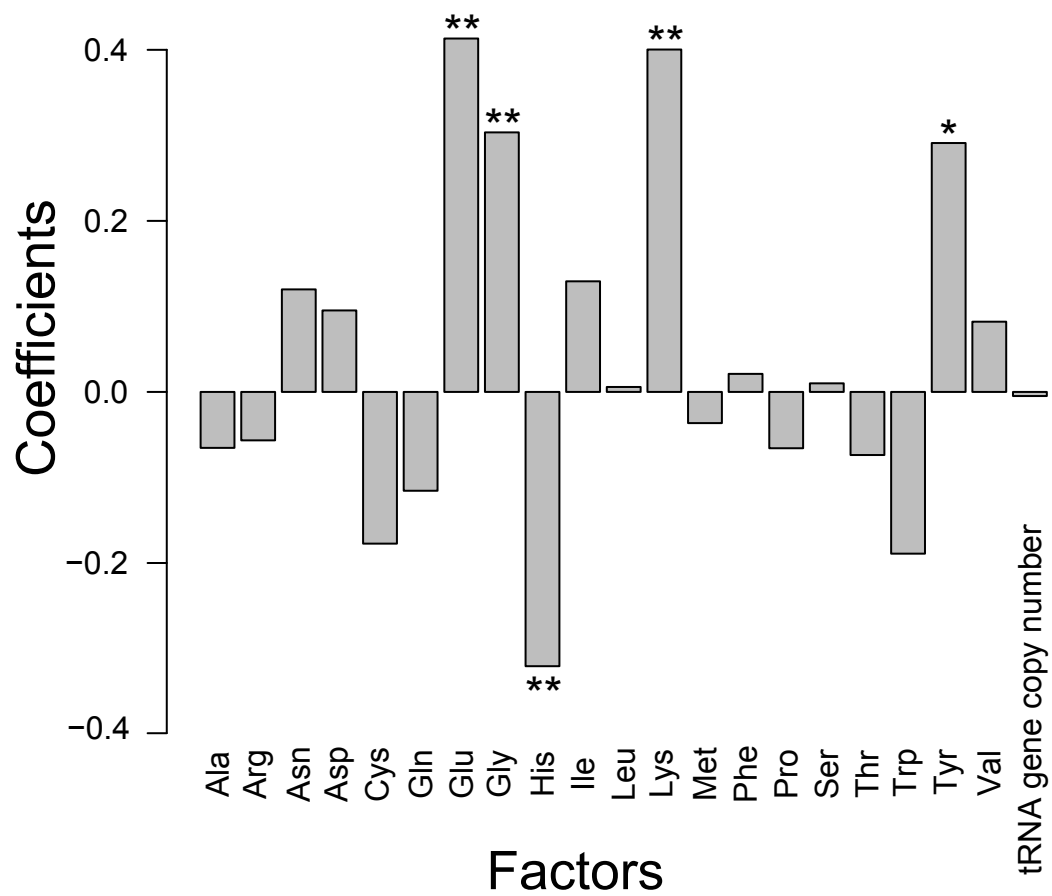

Figure S12

Supplement: Figure S12 — Significantly different CSTs among different amino acids. To quantify potential variations in CST among amino acids and among synonymous codons, we linearly regressed the CSTs of the 61 sense codons using the formula of , where CSTij is the CST of the jth codon of the ith amino acid, ai is the effect of amino acid i, b is the coefficient for the tRNA effect, tij is the gene copy number for the cognate tRNA of the jth codon of the ith amino acid, c is a constant equal to the mean CST of all sense codons, and ε is the residual effect. The parameters in the above linear regression were estimated by the least squares method. Asterisks indicate a statistically significant effect (*, nominal P<5%; **, nominal P<1%). Note the lack of a significant effect of the cognate tRNA gene copy number on the variation of synonymous CSTs, consistent with the results in Figure 1. (PDF) [file pgen.1002603.s012.pdf]
