# Supplementary material for: Balanced Codon Usage Optimizes Eukaryotic Translational Efficiency
Source: PLoS Genet. 2012 Mar 29;8(3):e1002603. doi: 10.1371/journal.pgen.1002603 (PMC3315465; doi:10.1371/journal.pgen.1002603)
Supplement: Figure S5 — Alignment of the DNA sequences of the four synonymous versions of mCherry used in our yeast experiments. Invariant sites among the four sequences are marked with asterisks. (PDF) [file pgen.1002603.s005.pdf]

```
mCherry version 1     ATG GTT TCA AAG GGC GAA GAA GAC AAT ATG GCT ATT ATT AAG GAA TTC ATG AGA TTT AAA
mCherry version 2     ATG GTT TCA AAG GGC GAA GAA GAC AAT ATG GCT ATT ATT AAG GAA TTC ATG AGA TTC AAG
mCherry version 3     ATG GTT TCA AAG GGC GAA GAA GAC AAT ATG GCT ATT ATT AAG GAA TTC ATG AGA TTT AAA
mCherry version 4     ATG GTT TCA AAG GGC GAA GAA GAC AAT ATG GCT ATT ATT AAG GAA TTC ATG AGA TTC AAG
                      *** *** *** *** *** *** *** *** *** *** *** *** *** *** *** *** *** *** **  **

mCherry version 1     GTA CAT ATG GAG GGA AGT GTA AAT GGA CAT GAG TTT GAG ATA GAG GGC GAG GGA GAA GGA
mCherry version 2     GTA CAT ATG GAA GGT AGT GTT AAT GGT CAT GAG TTC GAA ATA GAA GGT GAA GGT GAA GGT
mCherry version 3     GTT CAT ATG GAG GGT AGT GTT AAC GGT CAC GAA TTC GAA ATC GAA GGA GAA GGT GAA GGT
mCherry version 4     GTC CAC ATG GAA GGT TCT GTC AAC GGT CAC GAA TTC GAA ATT GAA GGT GAA GGT GAA GGT
                      **  **  *** **  **    * **  **  **  **  **  **  **  **  **  **  **  **  *** **

mCherry version 1     CGG CCG TAT GAG GGA ACG CAG ACG GCA AAA CTC AAA GTA ACG AAA GGA GGA CCG CTC CCG
mCherry version 2     AGA CCA TAT GAA GGT ACG CAA ACG GCT AAG CTC AAG GTA ACC AAG GGT GGT CCA CTC CCA
mCherry version 3     AGA CCA TAC GAG GGT ACC CAA ACT GCT AAG TTG AAA GTC ACA AAG GGT GGG CCT TTA CCA
mCherry version 4     AGA CCA TAC GAA GGT ACC CAA ACC GCT AAG TTG AAG GTC ACC AAG GGT GGT CCA TTG CCA
                       *  **  **  **  **  **  **  **  **  **   *  **  **  **  **  **  **  **   *  **

mCherry version 1     TTT GCA TGG GAT ATA CTC AGT CCG CAG TTT ATG TAT GGA AGT AAA GCA TAT GTA AAA CAT
mCherry version 2     TTC GCT TGG GAT ATA CTC AGT CCA CAG TTC ATG TAT GGT AGT AAG GCA TAT GTA AAG CAT
mCherry version 3     TTT GCC TGG GAC ATT TTG TCT CCA CAA TTC ATG TAT GGT TCC AAA GCT TAC GTG AAG CAT
mCherry version 4     TTC GCT TGG GAT ATT TTG TCT CCA CAA TTC ATG TAC GGT TCT AAG GCT TAC GTC AAG CAC
                      **  **  *** **  **   *    * **  **  **  *** **  **      **  **  **  **  **  **

mCherry version 1     CCG GCA GAT ATA CCG GAT TAT CTC AAA CTC AGT TTT CCG GAG GGA TTT AAA TGG GAG CGG
mCherry version 2     CCG GCA GAT ATA CCG GAT TAT CTC AAG TTG AGT TTC CCA GAA GGT TTC AAG TGG GAA CGG
mCherry version 3     CCT GCA GAT ATC CCA GAT TAT CTA AAA TTG TCT TTT CCA GAA GGT TTC AAG TGG GAA AGG
mCherry version 4     CCA GCT GAT ATT CCA GAT TAC TTG AAG TTG TCT TTC CCA GAA GGT TTC AAG TGG GAA AGA
                      **  **  *** **  **  *** **   *  **   *    * **  **  **  **  **  **  *** **   *

mCherry version 1     GTA ATG AAT TTT GAG GAT GGA GGA GTA GTA ACG GTA ACG CAG GAT AGT AGT CTC CAG GAT
mCherry version 2     GTA ATG AAT TTC GAG GAT GGT GGT GTA GTA ACG GTG ACG CAG GAT AGT AGT CTC CAA GAT
mCherry version 3     GTT ATG AAT TTT GAA GAC GGT GGT GTC GTA ACC GTT ACT CAA GAT TCA TCT TTA CAG GAT
mCherry version 4     GTC ATG AAC TTC GAA GAT GGT GGT GTC GTC ACC GTC ACC CAA GAT TCT TCT TTG CAA GAT
                      **  *** **  **  **  **  **  **  **  **  **  **  **  **  ***       *  *  **  ***

mCherry version 1     GGA GAG TTT ATA TAT AAA GTA AAA CTC CGG GGA ACG AAT TTT CCG AGT GAT GGA CCG GTA
mCherry version 2     GGT GAG TTC ATA TAC AAG GTA AAG TTG CGG GGT ACG AAC TTC CCA AGT GAT GGT CCA GTA
mCherry version 3     GGC GAA TTC ATA TAC AAA GTC AAG TTG AGA GGT ACG AAC TTC CCC TCC GAC GGT CCA GTT
mCherry version 4     GGT GAA TTC ATT TAC AAG GTC AAG TTG AGA GGT ACC AAC TTC CCA TCT GAT GGT CCA GTC
                      **  **  **  **  **  **  **  **   *   *  **  **  **  **  **      **  **  **  **

mCherry version 1     ATG CAG AAA AAA ACG ATG GGA TGG GAG GCA AGT AGT GAG CGG ATG TAT CCG GAG GAT GGA
mCherry version 2     ATG CAA AAG AAG ACG ATG GGT TGG GAA GCA AGT TCT GAG CGG ATG TAT CCG GAG GAT GGT
mCherry version 3     ATG CAA AAA AAG ACC ATG GGT TGG GAG GCC TCT AGC GAA AGA ATG TAT CCA GAA GAT GGT
mCherry version 4     ATG CAA AAG AAG ACC ATG GGT TGG GAA GCT TCT TCT GAA AGA ATG TAC CCA GAA GAT GGT
                      *** **  **  **  **  *** **  *** **  **    *     **   *  *** **  **  **  *** **

mCherry version 1     GCA CTC AAA GGA GAG ATA AAA CAG CGG CTC AAA CTC AAA GAT GGA GGA CAT TAT GAT GCA
mCherry version 2     GCA CTC AAG GGT GAG ATA AAG CAG AGA CTC AAG CTC AAG GAT GGT GGT CAT TAT GAT GCA
mCherry version 3     GCT CTG AAA GGA GAA ATC AAG CAA CGT TTG AAA TTA AAG GAT GGT GGT CAC TAC GAC GCT
mCherry version 4     GCT TTG AAG GGT GAA ATT AAG CAA AGA TTG AAG TTG AAG GAT GGT GGT CAC TAC GAT GCT
                      **   *  **  **  **  **  **  **   *   *  **   *  **  *** **  **  **  **  **  **

mCherry version 1     GAG GTA AAA ACG ACG TAT AAA GCA AAA AAA CCG GTA CAG CTC CCG GGA GCA TAT AAT GTA
mCherry version 2     GAG GTA AAG ACG ACG TAT AAG GCT AAG AAG CCG GTC CAG TTG CCG GGT GCA TAC AAT GTA
mCherry version 3     GAA GTT AAA ACT ACA TAT AAG GCC AAA AAG CCT GTC CAA TTG CCA GGT GCA TAC AAC GTT
mCherry version 4     GAA GTC AAG ACC ACC TAC AAG GCT AAG AAG CCA GTC CAA TTG CCA GGT GCT TAC AAC GTC
                      **  **  **  **  **  **  **  **  **  **  **  **  **   *  **  **  **  **  **  **

mCherry version 1     AAT ATA AAA CTC GAT ATA ACG AGT CAT AAT GAG GAT TAT ACG ATA GTA GAG CAG TAT GAG
mCherry version 2     AAT ATA AAG TTG GAT ATT ACC TCT CAT AAT GAG GAT TAT ACG ATA GTA GAA CAG TAT GAA
mCherry version 3     AAT ATT AAG CTT GAT ATC ACC TCT CAT AAC GAA GAT TAT ACT ATT GTC GAG CAA TAC GAA
mCherry version 4     AAC ATT AAG TTG GAT ATT ACC TCT CAC AAC GAA GAT TAC ACC ATT GTC GAA CAA TAC GAA
                      **  **  **   *  *** **  **    * **  **  **  *** **  **  **  **  **  **  **  **

mCherry version 1     CGG GCA GAG GGA CGG CAT AGT ACG GGA GGA ATG GAT GAG CTC TAT AAA
mCherry version 2     AGA GCA GAA GGT CGG CAT TCT ACC GGT GGT ATG GAT GAG CTC TAT AAG
mCherry version 3     AGA GCT GAA GGT AGA CAC TCC ACT GGC GGT ATG GAC GAA TTG TAC AAG
mCherry version 4     AGA GCT GAA GGT AGA CAC TCT ACC GGT GGT ATG GAT GAA TTG TAC AAG
                       *  **  **  **   *  **      **  **  **  *** **  **   *  **  **
```

	CAI	D_{ncu}
mCherry version 1	0.07	0.81
mCherry version 2	0.24	0.46
mCherry version 3	0.53	0.02
mCherry version 4	0.93	0.37

Figure S5
