# Supplementary material for: Balanced Codon Usage Optimizes Eukaryotic Translational Efficiency
Source: PLoS Genet. 2012 Mar 29;8(3):e1002603. doi: 10.1371/journal.pgen.1002603 (PMC3315465; doi:10.1371/journal.pgen.1002603)
Supplement: Figure S1 — The procedure for estimating codon selection times (CSTs) from ribosome profiling data. Circled numbers correspond to the equations in Materials and Methods and thick arrows show the iterations. (PDF) [file pgen.1002603.s001.pdf]

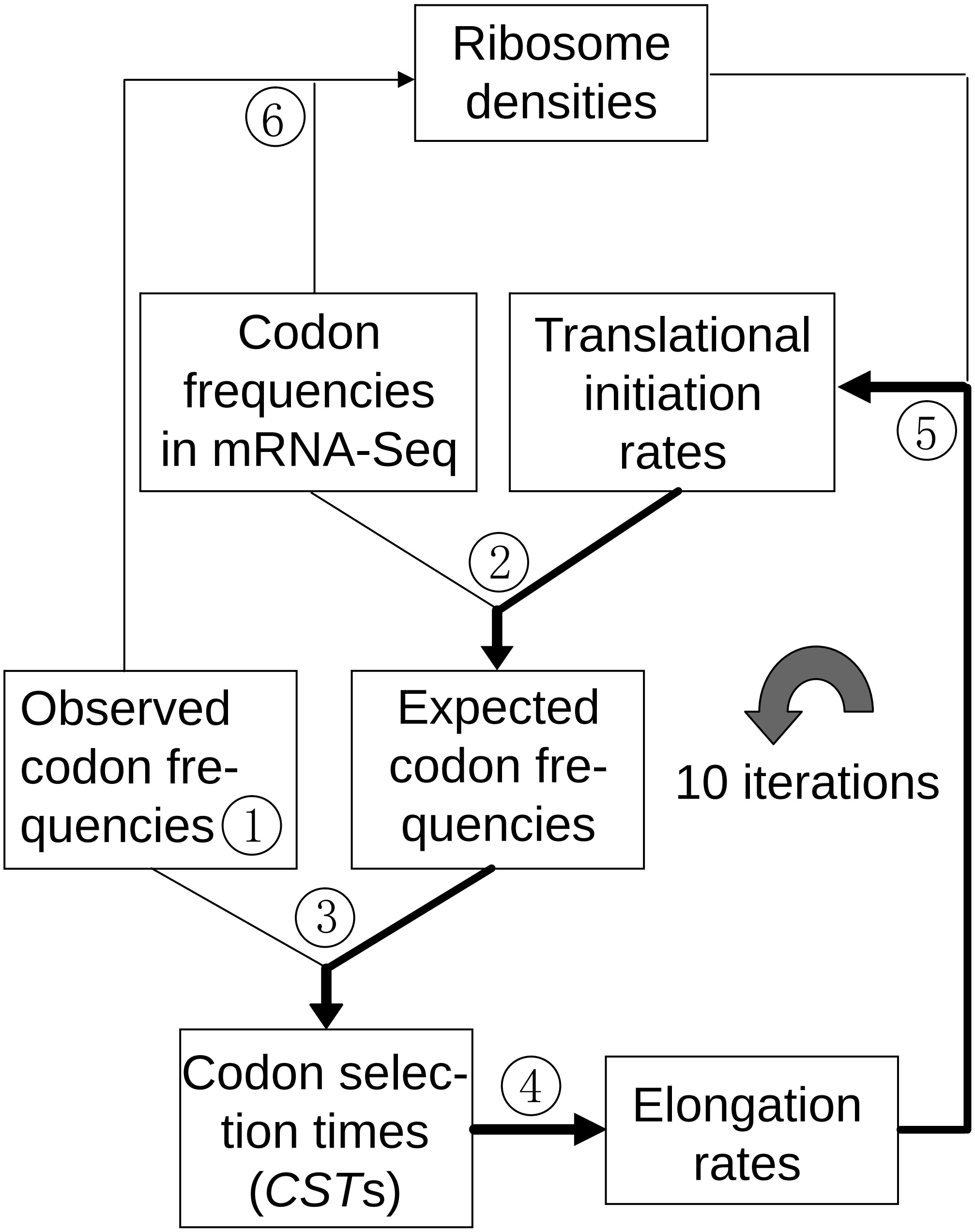

Ribosome densities
⑥
Codon frequencies in mRNA-Seq
Translational initiation rates
⑤
②
Observed codon fre- quencies ①
Expected codon fre- quencies
10 iterations
③
Codon selec- tion times (*CST*s)
④
Elongation rates

Figure S1
