# Supplementary material for: Balanced Codon Usage Optimizes Eukaryotic Translational Efficiency
Source: PLoS Genet. 2012 Mar 29;8(3):e1002603. doi: 10.1371/journal.pgen.1002603 (PMC3315465; doi:10.1371/journal.pgen.1002603)
Supplement: Table S2 — Unequal use of iso-synonymous codons in S. cerevisiae. (PDF) [file pgen.1002603.s014.pdf]

Table S2. Unequal use of iso-synonymous codons in *S. cerevisiae*.

| Iso-synonymous codon groups[1] | Codon | tRNA | aa | Perfect match[2] | Stability[3] | *RSCU'* |
|---|---|---|---|---|---|---|
| 1* | GCT | AGC | Ala | 1 | 1 | 0.717 |
| | GCC | AGC | Ala | 0 | 0 | 0.248 |
| 2* | GCA | TGC | Ala | 1 | 1 | 0.030 |
| | GCG | TGC | Ala | 0 | 0 | 0.005 |
| 3* | CGT | ACG | Arg | 1 | 1 | 0.174 |
| | CGC | ACG | Arg | 0 | 0 | 0.004 |
| | CGA | ACG | Arg | 0 | 1 | 0.003 |
| 4* | AAT | GTT | Asn | 0 | 0 | 0.147 |
| | AAC | GTT | Asn | 1 | 1 | 0.853 |
| 5* | GAT | GTC | Asp | 0 | 1 | 0.430 |
| | GAC | GTC | Asp | 1 | 1 | 0.570 |
| 6+ | TGT | GCA | Cys | 0 | 1 | 0.868 |
| | TGC | GCA | Cys | 1 | 1 | 0.132 |
| 7 | GGT | GCC | Gly | 0 | 1 | 0.929 |
| | GGC | GCC | Gly | 1 | 0 | 0.047 |
| 8* | CAT | GTG | His | 0 | 1 | 0.263 |
| | CAC | GTG | His | 1 | 1 | 0.737 |
| 9 | ATT | AAT | Ile | 1 | 0 | 0.457 |
| | ATC | AAT | Ile | 0 | 1 | 0.519 |
| 10+ | CTT | GAG | Leu | 0 | 1 | 0.026 |
| | CTC | GAG | Leu | 1 | 1 | 0.005 |
| 11* | CTA | TAG | Leu | 1 | 1 | 0.071 |
| | CTG | TAG | Leu | 0 | 1 | 0.008 |
| 12* | TTT | GAA | Phe | 0 | 0 | 0.194 |
| | TTC | GAA | Phe | 1 | 1 | 0.806 |
| 13* | CCT | AGG | Pro | 1 | 1 | 0.123 |
| | CCC | AGG | Pro | 0 | 0 | 0.011 |
| 14* | CCA | TGG | Pro | 1 | 1 | 0.857 |
| | CCG | TGG | Pro | 0 | 0 | 0.009 |
| 15* | TCT | AGA | Ser | 1 | 1 | 0.520 |
| | TCC | AGA | Ser | 0 | 1 | 0.347 |
| 16+ | AGT | GCT | Ser | 0 | 1 | 0.041 |
| | AGC | GCT | Ser | 1 | 1 | 0.027 |
| 17* | ACT | AGT | Thr | 1 | 1 | 0.507 |
| | ACC | AGT | Thr | 0 | 1 | 0.442 |
| 18* | TAT | GTA | Tyr | 0 | 0 | 0.138 |
| | TAC | GTA | Tyr | 1 | 1 | 0.862 |
| 19* | GTT | AAC | Val | 1 | 1 | 0.549 |
| | GTC | AAC | Val | 0 | 1 | 0.416 |

[1] Iso-synonymous codons are recognized by the same tRNA species. The perfect-match rule states that perfect codon-anticodon matches are favored over imperfect matches. The stability rule assumes that a codon-anticodon interaction is stable if the codon has either 1 or 2 G/C and is otherwise unstable (Rocha 2004 *Genome Res* **14,** 2279). This rule claims that codons with stable interactions with anticodons are preferred. We denote a star (correct prediction) or cross (incorrect prediction) for cases where the predictions of the two rules are compatible with each other.
[2] Perfect codon-anticodon matches are denoted by 1 and imperfect matches are denoted by 0.
[3] Stable codon-anticodon interactions are denoted by 1 and unstable interactions are denoted by 0.
