# Supplementary material for: Balanced Codon Usage Optimizes Eukaryotic Translational Efficiency
Source: PLoS Genet. 2012 Mar 29;8(3):e1002603. doi: 10.1371/journal.pgen.1002603 (PMC3315465; doi:10.1371/journal.pgen.1002603)
Supplement: Table S1 — Euclidian and Manhattan distances between the observed tRNA fractions and the predictions of the proportional rule, square rule, and truncation rule, respectively. (PDF) [file pgen.1002603.s013.pdf]

Table S1. Euclidian and Manhattan distances between the observed tRNA fractions and the predictions of the proportional rule, square rule, and truncation rule, respectively.

Transcriptomes	Euclidian distance (*P* value[1])			Manhattan distance (*P* value[1])		
	Proportional	Square	Truncation	Proportional	Square	Truncation
S. cerevisiae	0.42 ($<1\times10^{-6}$)	0.90 (1×10^{-6})	1.45 ($<1\times10^{-6}$)	1.91 ($<1\times10^{-6}$)	4.49 (1×10^{-5})	7.29 ($<1\times10^{-6}$)
S. pombe	0.44 ($<1\times10^{-6}$)	0.62 ($<1\times10^{-6}$)	1.56 ($<1\times10^{-6}$)	2.22 ($<1\times10^{-6}$)	3.06 ($<1\times10^{-6}$)	7.66 ($<1\times10^{-6}$)
A. thaliana	0.84 ($<1\times10^{-6}$)	1.18 ($<1\times10^{-6}$)	2.23 (2.4×10^{-5})	3.55 ($<1\times10^{-6}$)	4.68 ($<1\times10^{-6}$)	11.78 (1.2×10^{-5})
C. elegans	0.49 ($<1\times10^{-6}$)	0.71 ($<1\times10^{-6}$)	1.65 (2×10^{-6})	2.44 ($<1\times10^{-6}$)	3.47 ($<1\times10^{-6}$)	8.41 (7×10^{-6})
D. melanogaster	0.43 ($<1\times10^{-6}$)	0.66 ($<1\times10^{-6}$)	1.83 ($<1\times10^{-6}$)	1.95 ($<1\times10^{-6}$)	3.25 ($<1\times10^{-6}$)	9.41 ($<1\times10^{-6}$)
M. musculus	0.60 ($<1\times10^{-6}$)	0.67 ($<1\times10^{-6}$)	2.20 (5×10^{-5})	2.83 ($<1\times10^{-6}$)	3.12 ($<1\times10^{-6}$)	11.4 (5×10^{-5})
H. sapiens	0.49 ($<1\times10^{-6}$)	0.51 ($<1\times10^{-6}$)	2.03 ($<1\times10^{-6}$)	2.42 ($<1\times10^{-6}$)	2.51 ($<1\times10^{-6}$)	10.27 ($<1\times10^{-6}$)

[1] *P* values indicate the probability that a distance generated by random tRNA pool is smaller than the observed distance, determined by 10^6 simulations.
